# Chemical Vapor Deposition of Monolayer Graphene on Centimeter-Sized Cu(111) for Nanoelectronics Applications

**DOI:** 10.1021/acsanm.5c00588

**Published:** 2025-02-24

**Authors:** Jia Tu, Wentong Zhou, Amin Kiani, Lawrence M. Wolf, Mingdi Yan

**Affiliations:** Department of Chemistry, University of Massachusetts Lowell, Lowell, Massachusetts 01854, United States

**Keywords:** Cu(111), polycrystalline Cu, strain-free abnormal grain growth, graphene, chemical vapor deposition

## Abstract

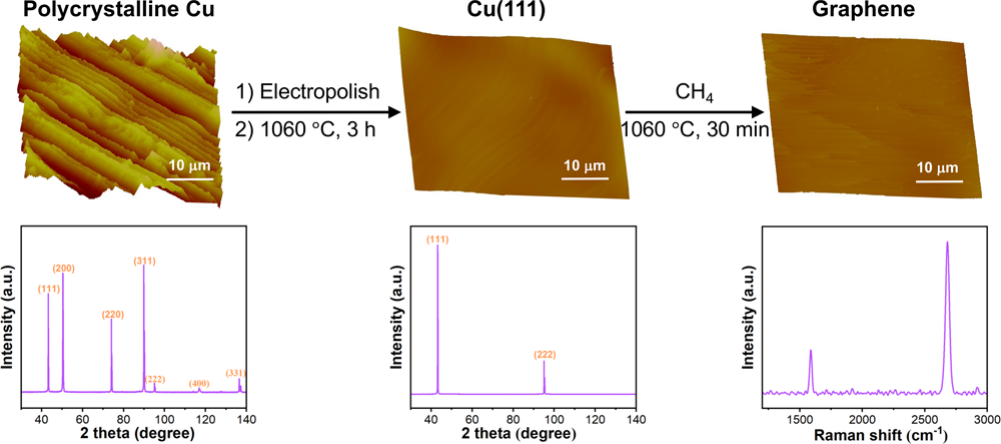

We report a fast and straightforward preparation of centimeter-sized Cu(111) from polycrystalline Cu foil by the strain-free abnormal grain growth method and the subsequent growth of monolayer graphene by chemical vapor deposition (CVD). The fabrication of Cu(111) and graphene was streamlined into a straightforward process using a CVD system consisting of a tube furnace and a quartz boat. It was found that the annealing temperature and time are critical in the growth of Cu(111). Heating at 1060 °C for 3 h led to the conversion of polycrystalline Cu to Cu(111), yielding large grains and high-quality monolayer graphene. Molecular dynamics (MD) simulations supported the experimental findings, demonstrating that annealing leads to increased mobility of Cu grain boundaries. MD simulations further revealed that Cu(111) in the polycrystalline Cu serves as the seed for the growth of large Cu(111) grains. The impact of mechanical deformation on the conversion polycrystalline Cu to Cu(111) was also investigated, showing that a flat and nondeformed Cu foil was essential for the growth of large Cu(111) grains. The deformed areas could not be fully converted to Cu(111), a result which was further supported by MD simulations. Finally, the issue of Cu foil adhering to the quartz boat could be solved by flushing the CVD system with the working gases prior to annealing. The streamlined fabrication process for Cu(111) and monolayer graphene offers broad potential for applications such as nanoelectronics.

## Introduction

Cu is widely used as the substrate for the preparation of graphene by chemical vapor deposition (CVD).^1−3^ The low solubility of carbon in the bulk Cu enables the growth of monolayer single crystal graphene through a self-limited growth process. Additionally, the low cost of Cu makes the mass production of graphene a reality.^4^ The quality of CVD graphene is closely associated with the crystal orientation and purity of the Cu substrate. Polycrystalline Cu causes random nucleation, which leads to the formation of polycrystalline graphene of different orientations and grain boundaries when domains coalesce,^5−7^ which decreases the electron transport and mechanical strength of graphene.^5,8−10^ Among the various crystalline facets of Cu, low-index facets promote the rapid growth of monolayer graphene having few defects, with Cu(111) producing the highest quality monolayer graphene with high area coverage.^11^ Furthermore, Cu(111) and graphene possess the same *C*_3_ rotation symmetry element. As a result, the lattice match between Cu(111) and graphene is high, with only a small mismatch of 3–4%.^12,13^ This lattice match promotes aligned growth of graphene on Cu(111), resulting in a significant reduction in the density of grain boundaries.^11,12,14−20^

The metal substrate can also influence the electronic properties and chemical reactivity of graphene. Studies have shown that the electronic properties of graphene can be substantially changed when it is adsorbed on a metal substrate.^21−23^ For example, the strong interaction between graphene and Ni(111) leads to orbital hybridization, charge redistribution, band gap opening, and the lengthening of the carbon–carbon bond.^21,22^ We have demonstrated that the chemical reactivity of graphene toward cycloaddition reactions can be enhanced by a metal substrate.^23−25^ However, while polycrystalline metal substrates were used in the experiments, single crystal metals were used in computations. The availability of single crystal metal substrates such as Cu(111) would bring experimental results more in line with theoretical predictions. Nonetheless, commercial Cu(111) is costly and primarily available in millimeter sizes, whereas polycrystalline Cu foils are inexpensive and are available in meter sizes.

Two methods are generally employed to prepare single crystal metal films or foils: epitaxial deposition and grain growth. Epitaxial deposition can be achieved through either physical deposition of vaporized metal onto a substrate, or the reduction of metal ions in solution by electrodeposition onto a conducting or semiconducting substrate.^1^ For the fabrication of single crystal Cu(111) by epitaxial deposition, Cu films are normally deposited on inorganic single crystal substrates such as C-plane sapphire(0001), MgO(111), or MgAl_2_O_4_(111) by magnetron sputtering.^3,16,17,19,26−29^ Several drawbacks limit the wide use of this method for the synthesis of graphene. First, the size of graphene fabricated is limited to the size of the underlying substrate, which may not meet the requirement of mass production.^1,30^ Second, the inorganic substrates require pretreatment, including cleaning, annealing with oxygen, and postannealing for up to 12 h, which is time-consuming.^3^ Finally, transferring graphene from such substrates is challenging. While Cu can be removed readily by acid etching, other inorganic substrates are not as easily removable.

The grain growth method for the preparation of single crystal metals involves annealing polycrystalline metals at high temperatures. During this process, atoms rearrange into a lower energy configuration, driven by the minimization of energy stored in the form of grain boundaries that are thermodynamically unstable at high temperatures. There are two types of grain growth: normal grain growth and abnormal grain growth.^31^ In normal grain growth, grains grow homogeneously into a narrow grain size distribution and grain shape, which generally produces a maximum grain size of 2–3 times the thickness of the metal film. In abnormal grain growth, grains grow in a heterogeneous manner, where larger grains grow by consuming smaller grains. This process can result in single crystal metals of much larger sizes. The abnormal grain growth method is preferred for the preparation of single crystal Cu(111), which is the ideal substrate for the fabrication of large area high-quality graphene.

Different protocols have been developed for the preparation of Cu(111) foils by abnormal grain growth (Table S1). For example, Park and co-workers annealed Cu foils in Ar/H_2_ at 1030 °C for up to 12 h to yield ∼95% Cu(111).^32^ Liu et al. fabricated 5 cm × 50 cm Cu(111) foil by continuously sliding the foil in the furnace at 500 sccm Ar and 1030 °C to create a temperature gradient which promoted the movement of grain boundaries.^13^ The Ruoff group developed a contact-free method by suspending a polycrystalline Cu foil on a quartz holder to reduce interfacial stress, followed by annealing at 1050 °C for 12–18 h.^9,30^ Reckinger et al.,^33^ and more recently, Liu and co-workers,^34^ reported that a pretreatment of polycrystalline Cu foils by low temperature oxidation to Cu_*x*_O induced rapid formation of Cu(111) upon annealing in hydrogen gas. In contrast, Liu and co-workers found that oxidation of Cu foils prior to annealing yielded high-index Cu facets, and only Cu foils without preoxidation could be converted to Cu(111) upon annealing.^35^ It is evident that despite the development of various protocols, the preparation of Cu(111) from polycrystalline Cu foils is not as straightforward as it may seem. The interplay of different experimental parameters is not yet fully understood.

**Scheme 1:**
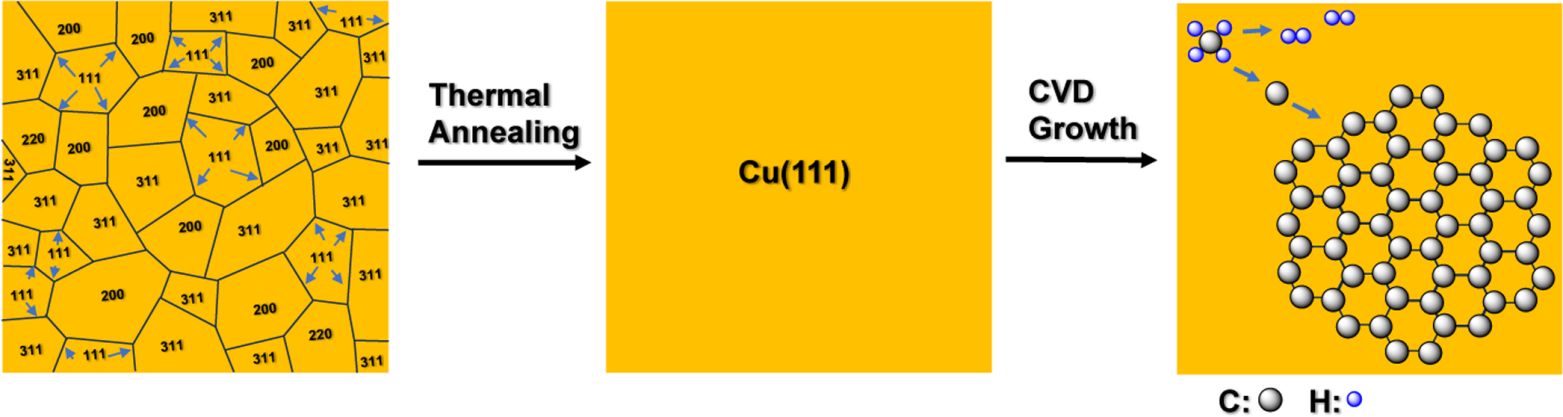
Streamlined Preparation of Cu(111) from Polycrystalline Cu Foil through Thermal Annealing and the Subsequent Growth of Monolayer Graphene by CVD

In this work, we developed a straightford process to prepare Cu(111) from commercial polycrystalline Cu foils via strain-free abnormal grain growth mechanism and to subsequently grow high-quality monolayer graphene directly on the Cu(111) foil (Scheme 1). The two steps were integrated into a streamlined process, carried out in a home-built CVD setup consisting of a Lindberg/Blue M tube furnace that is frequently used in academic research laboratories. No additional special apparatus is needed, and high purity Cu(111) was obtained by annealing polycrystalline Cu foils at 1060 °C for 3 h. The impact of annealing temperature on crystal orientation and grain size of Cu foils were investigated by XRD, atomic force microscopy (AFM), optical microscopy, and scanning electron microscopy (SEM) and the results were supported by molecular dynamics (MD) simulations. The quality of graphene grown on different Cu substrates was examined by Raman spectroscopy, AFM and optical microscopy. The impact of deformation on the grain size of Cu(111) and quality of graphene was studied both experimentally and computationally. The highest quality graphene having no/low defects was obtained on electropolished nondeformed Cu foils annealed at 1060 °C for 3 h. Additionally, we discovered that by purging the CVD system with argon prior to annealing, the issue of evaporation and adhesion of the Cu foil to the quartz boat was completely eliminated. This simplified and streamlined fabrication process for Cu(111) and high-quality monolayer graphene may find a wide range of applications, including in nanoelectronics.

## Expriemental Section

### Materials and Instrumentation

The following materials were acquired from commercial sources, and used directly unless otherwise specified: polycrystalline Cu foil (0.025 mm thick, annealed, uncoated, 99.8%, Alfa Aesar), Cu plate (0.675 mm thick, annealed, 99.9%, Alfa Aesar), ethanol (200 proof, Decon Laboratories, Inc.), isopropyl alcohol (>99.5%, Honeywell and Fisher Chemical), phosphoric acid (>85 wt %, Honeywell), urea (99.63%, certified ACS Fisher Chemical, Fisher Scientific), hydrogen, argon, and methane gases (ultrahigh purity grade, Airgas), poly(methyl methacrylate) (PMMA, average molecular weight: 996,000, Aldrich Chemical), acetone and methanol (certified ACS, Fisher Chemical), hydrochloric acid (certified ACS Plus, Fisher Chemical), iron(III) chloride (97%, Sigma-Aldrich). Water (resistivity 18.2 MΩ·cm) was obtained from a Milli-Q ultrapure water system (MilliporeSigma).

XRD characterization was carried out on a Rigaku MiniFlex 600 X-ray diffractometer with Cu Kα (λ = 1.5418 Å) radiation. The 2θ range was 30–140°, and the scan rate was 10° min^–1^. AFM imaging was conducted on a Bruker Multimode AFM with a Nanoscope IV controller in the tapping mode. Optical microscopy images were collected on a Nikon ECLIPSE L200 microscope with 50× objective lens and a Moticam 2500 camera (5.0 M Pixel).

The Raman measurements were performed at room temperature using a Raman spectrometer (Bruker Senterra I) equipped with an Olympus BX optical microscope (50× objective lens) and a 532 nm laser. The incident power of 1 mW and irradiation time of 5 or 10 s were used unless otherwise noted. The Raman spectra were analyzed using an object-oriented Python analysis tool, which can efficiently parse multiple Raman spectra and perform additional tasks, including baseline correction, normalization, and peak feature detection. The baseline correction algorithm was doubly reweighted penalized least-squares (drPLS) developed by Yang and co-workers.^36^ Raman mapping was conducted on a Renishaw inVia Raman microscope equipped with a 532 nm laser line with laser intensity around 1.68 mW. SEM images were obtained on a JEOL 7401F field emission scanning electron microscope (accelerating voltage 10 kV).

### Electropolishing Cu Foils

A stock solution of the electropolishing solution was prepared from 1000 mL of Milli-Q water, 500 mL of phosphoric acid, 500 mL of ethanol, 100 mL of isopropyl alcohol, and 10.0 g of urea. The polycrystalline Cu foil was cut into 7 cm × 2 cm pieces by scissors, and washed with water, acetone, methanol, and ethanol. In the electropolishing setup, the polycrystalline Cu foil serves as the anode and the Cu plate serves as the sacrificial cathode (Figure S2). The power supply provided a constant current of 2.80 A, and the voltage fluctuated in the range of 6.0–7.2 V. After turning on the power supply for 2.5 min, the Cu foil was rinsed sequentially with Milli-Q water, ethanol, methanol, and acetone. It was then soaked in water and acetone separately for ∼1 min to further remove the residual reagents on the Cu surface. Finally, it was rinsed sequentially with water, methanol, ethanol, and acetone, and dried with argon.

### Fabrication of Cu(111)

The electropolished Cu foil was placed horizontally on a high-purity quartz boat (6 mL, 114 mm L × 18 mm W × 8.5 mm H, MSE Supplies). The quartz boat was then inserted into the center area of a 1″-diameter fused quartz tube (22 mm I.D. × 25 mm O.D. × 4 ft., Technical Glass Products Inc.) in a tube furnace (Thermo Scientific Lindberg/Blue M mini-mite TF55030 A-1). The base pressure in the tube was reduced by applying vacuum (Welch pump Model no. 1402B-01) to 195 mTorr as measured by a pressure gauge (KJL-6000Series, Kurt J. Lesker). The hydrogen, methane, and argon gas tanks were opened to purge the tube and to remove residual air in the system. The tube was then backfilled with hydrogen gas at 10 sccm (standard cubic centimeters per minute) and argon gas at 15–16 sccm. The inner pressure in the tube was maintained at 990–975 mTorr with fluctuation. The furnace was ramped to the desired temperature, e.g., 1060 °C, which took about 22 min. The electropolished Cu foil was then annealed at specified temperature and duration.

### Fabrication of Graphene

After annealing the Cu foil, the argon gas was turned off and methane (3–6 sccm, total pressure: 700–820 mTorr) was introduced into the quartz tube for 30 min to grow graphene. The furnace was cooled rapidly under flow of both hydrogen and methane, which took about 1 h 40 min to reach about 50 °C. Afterward, the gases were turned off, and the air valve was opened to the ambient atmosphere. The resulting graphene sample was stored in a clean plastic Petri dish.

The procedure of graphene grown on as-received polycrystalline Cu foil was similar to the graphene grown on Cu(111). The difference is that the as-received polycrystalline Cu foil was directly inserted into the fused quartz tube and the annealing was carried out at 1000 °C for about 1 h. Graphene was grown following the same procedure described above.

### Transfer of Graphene to Silicon Wafer^37^

A Silicon wafer having a 2800 Å oxide layer (single-side polished, Fuleda Technology) was cut into 0.8 cm × 0.8 cm pieces. The pieces were rinsed with ethanol, acetone, and water, followed by a scrub in ethanol with a cotton swab. Finally, they were washed again with ethanol, acetone, and water.

A solution of PMMA in acetone (40 mg/mL) was spin-coated at 1000 rpm for 1 min to form a protective film on graphene. The sample was placed in the etching solution (1 M FeCl_3_ in 3 M HCl) with the Cu side floating on the solution to etch the Cu foil. After about 30 s, the sample was taken out and the bottom of the Cu foil was wiped several times to remove the bottom graphene layer. The sample was then placed in the etching solution and was etched at room temperature for about 3 h. The sample became transparent, and the Cu foil was etched away. The graphene on the PMMA film was washed with 1 M HCl solution once followed by Milli-Q water three times. Then, a piece of silicon wafer was immersed into the solution to scoop the floating film onto the wafer. The sample was dried in the air for about 12 h until there was no observable water on the surface of the sample. It was then soaked in ∼53 °C acetone on a 70 °C hot plate for about 40 min. Finally, the sample was dried at room temperature in air.

### MD Simulations

MD simulations were performed by applying 2D periodic boundary conditions and using the isothermal–isobaric ensemble (NPT) to maintain constant pressure and temperature. The Nosé–Hoover thermostat was applied to fix the temperature. The atomic interactions were described through the Embedded Atom Method (EAM) potential.^38^ A wide temperature cycle was employed, ranging from 300 K to annealing temperatures, and back to 300 K, executed over a 4.8 ns period. Variable annealing temperatures range between 1000 and 1300 K. Estimations of migration rates in the normal direction of the grain boundary interface were computed at various temperatures. This was achieved by extracting atomic coordinate data from trajectory snapshots following a set of 15 simulations, each of which is with a unique set of random velocities. The pressure was kept at 0.1 bar in all three dimensions throughout the simulation. The time step for the simulation was set at 1 femtosecond (fs). The uniaxial tensile simulation was performed in the *x*-direction at a strain rate of 10^10^ s^–1^ with the previously provided *NPT* ensemble. All simulations were performed using the Lammps package (version 22 June 2022 update 1).^39^ All the initial models were built using the Atomsk package.^40^ Visualization and dislocation analysis were done using the Ovito Pro software.^41^

## Results

The fabrication of Cu(111) foils involves electropolishing polycrystalline Cu foils followed by annealing at high temperature in a reducing environment with hydrogen gas.

### Electropolishing Reduces Surface Roughness of Cu Foils

**Figure 1:**
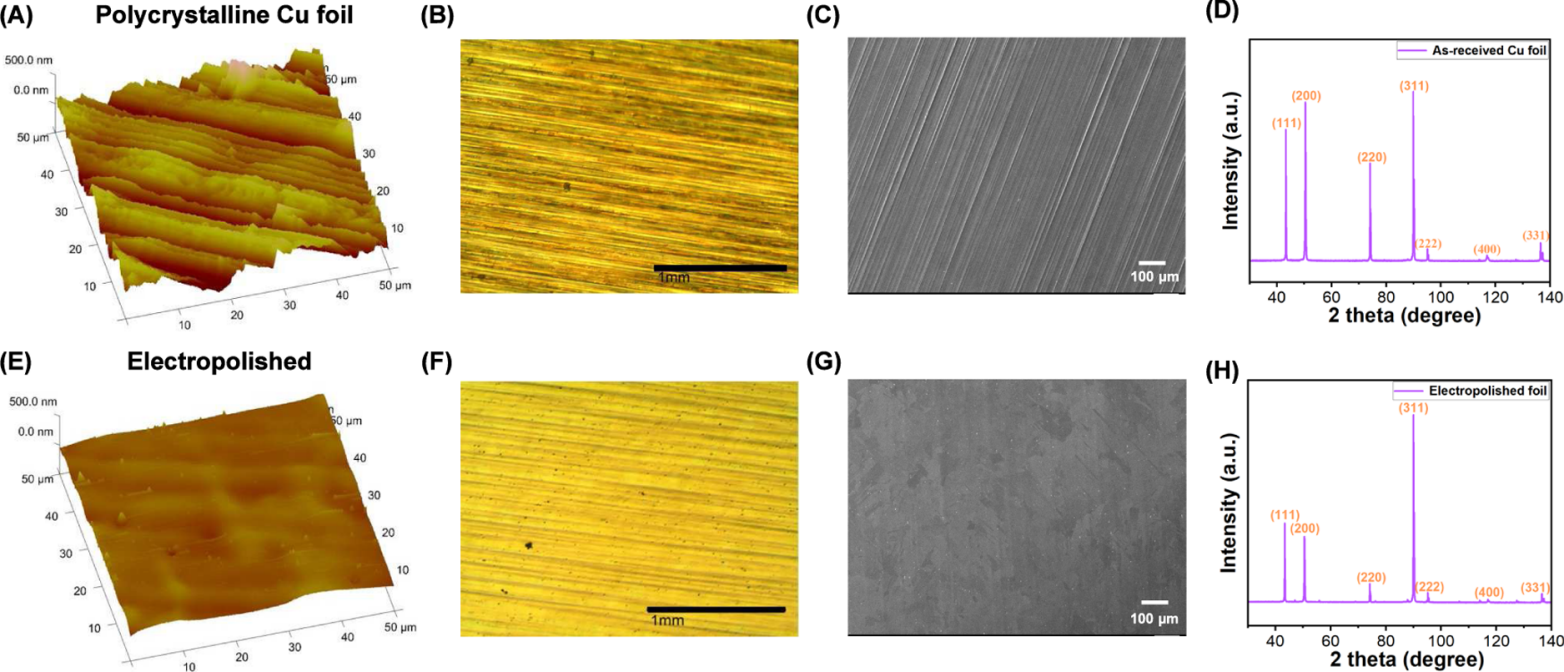
AFM images, optical microscopy, and SEM images, and XRD spectra of (A–D) as-received polycrystalline Cu foil, and (E–H) after electropolishing at the current of 2.80 A for 2.5 min. Additional AFM images of polycrystalline Cu foils before (Figure S1A–C) and after electropolishing at 2.80 A for 2.5 min (Figure S1D–F) or at 0.70 (Figure S3A–D) or 1.70 A (Figure S3E–H) for 2.5 min can be found in the Supporting Information.

As-received polycrystalline Cu foils are fairly rough, showing an average atomic force microscopy (AFM) root-mean-square (RMS) roughness of 113 ± 12 nm (Figures 1A, and S1A–C). The large elongated grooves, i.e., striations, are the result of mechanical processes such as rolling, while irregular grain boundaries and the orientation of individual grains within the foil further contribute to surface roughness (Figure 1B,C).^42^ To reduce the surface roughness, polycrystalline Cu foils were subjected to electropolishing, which is known to reduce striations and to smooth the surface of Cu foils.^43,44^ Electropolishing polycrystalline Cu foils at the current of 0.70 or 1.70 A for 2.5 min did not improve the surface roughness, giving an average RMS roughness of 153 ± 32 nm and 151 ± 43 nm, respectively (Figure S3). When the current was increased to 2.80 A, the RMS roughness decreased drastically to 38.8 ± 12 nm after electropolishing for 2.5 min (Figures 1E, and S1D–F). The decrease in surface roughness was evident also in the optical and SEM images of electropolished Cu foils (Figure 1F,G). A higher current was not attempted as the maximum allowable current of the power supply was 3.0 A.

The XRD spectrum of the polycrystalline Cu foil showed four dominant peaks at 90.0, 74.2, 50.5, and 43.3°, corresponding to (311), (220), (200), and (111) facets (JCPDS no. 04-0836), respectively (Figure 1D). After electropolishing, diffraction from the (311) facet became the dominant. The intensity of (111), (200), and (220) relative to that of (311) decreased from 78%, 94%, and 59% (Figure 1D) to 44%, 37%, and 12% (Figure 1H), with the (200) and (220) decreasing more than (111). Since electropolishing removes the surface layer, these results indicate that (200) and (220) dominate the surface while the bulk of the polycrystalline Cu foils is dominated by (311).

### Fabrication of Single Crystalline Cu(111) from Polycrystalline Cu Foils by Annealing

**Figure 2:**
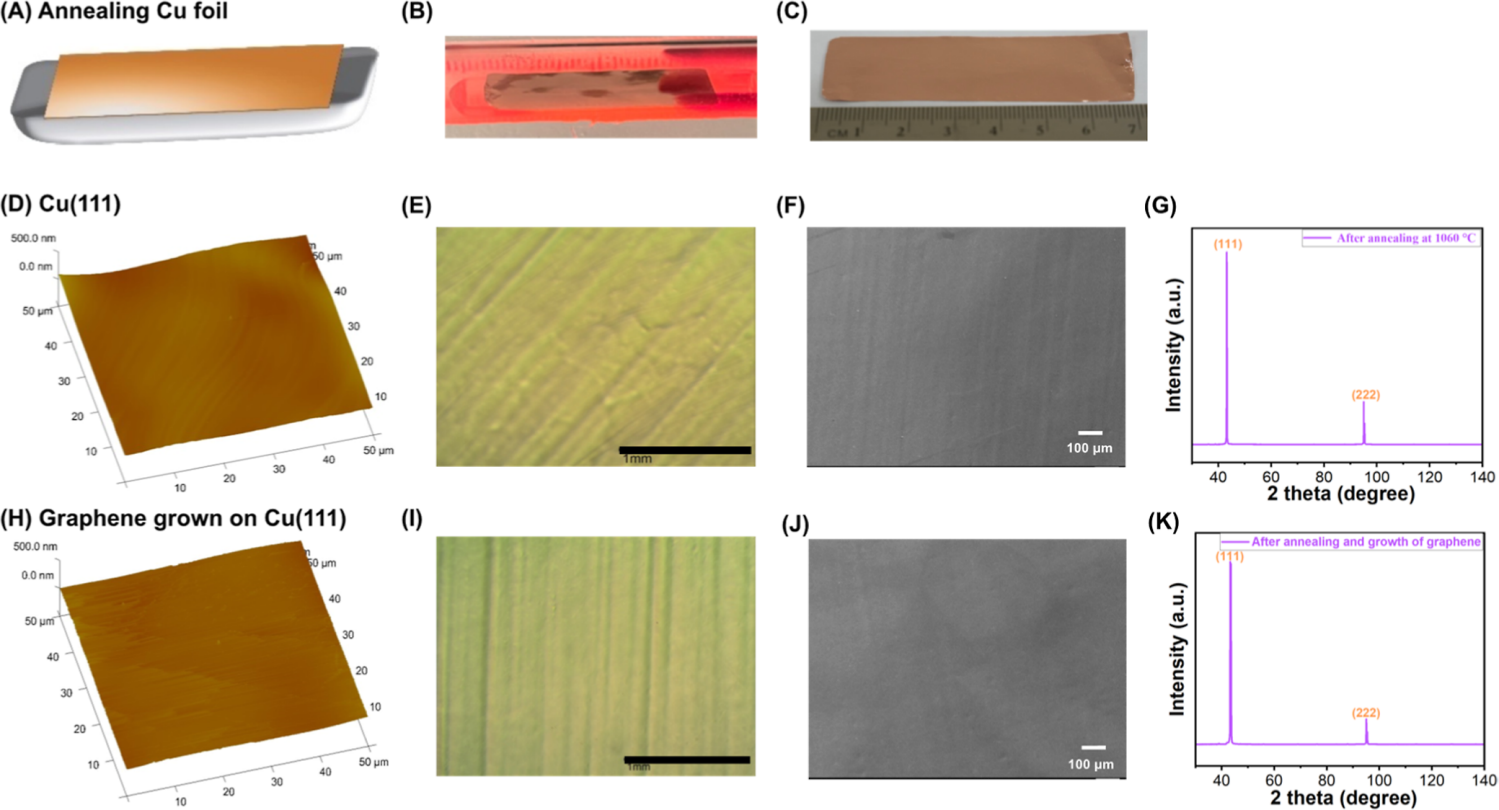
(A) Schematic of a Cu foil placed on a quartz boat. (B) Photograph of a Cu foil placed on the quartz boat inside the quartz tube after annealing at 1060 °C for 3 h. (C) Photograph of the annealed Cu foil after cooling to room temperature. (D) AFM image, (E) optical microscopy image, (F) SEM image, and (G) XRD spectrum of Cu(111) obtained by annealing electropolished polycrystalline Cu foil at 1060 °C for 3 h. (H) AFM image, (I) optical microscopy image, (J) SEM image, and (K) XRD spectrum of graphene grown on Cu(111).

Our CVD system is built from a tube furnace fitted with a 1″-diameter quartz tube that is too small to accommodate an additional sample holder. Since the quartz boat is used as the support for the Cu foil in the fabrication of graphene, it would be convenient if the quartz boat could be used for the fabrication of Cu(111) as well. To test this approach, we attempted different ways to place the Cu foil on the quartz boat with the goal of minimizing strain and maximizing the strain-free area of the Cu foil. We found that Cu foil placed horizontally on top of the quartz boat yielded the best results (Figure 2A). Figure 2B shows a 7 cm × 2 cm electropolished polycrystalline Cu foil on the quartz boat after annealing at 1060 °C for 3 h inside the furnace and after cooling to room temperature (Figure 2C). The surface smoothness further improved after annealing, showing a decrease in AFM RMS to 21.0 ± 4.2 nm (Figures 2D, and S1G–I) and reduced striations in optical and SEM images (Figures 2E,F, and S4A–H). The reduction in the RMS during the annealing process may be due to several reasons: (1) grain growth. During annealing, the grains in the polycrystalline Cu foils undergo abnormal grain growth, resulting in larger and more uniform grains. This process can help reduce surface roughness and enhance surface smoothness.^45^ (2) Surface reconstruction. The high temperature annealing can promote surface reconstruction of the Cu foil, leading to less corrugation and removal of dense step bunches and thermal grooves.^45,46^ (3) Removal of surface contamination. Surface contaminants and adsorbates contribute to surface roughness. Annealing at high temperature facilitates the removal of these contaminants, which can result in a smoother surface.^45^

The polycrystalline Cu completely converted to Cu(111) after annealing. A sharp (111) peak was observed, and the (311), (200) and (220) peaks completely disappeared (Figure 2G). The (222) facet, which has a 2θ value of 95.2° and is identical to the (111) facet,^47^ was present in some samples. Since no special apparatus or an additional holder is needed, this setup streamlined the fabrication of both Cu(111) and graphene into a simplified process.

### Annealing Temperature and Time are Critical in Producing Higher Quality Cu(111)

**Figure 3:**
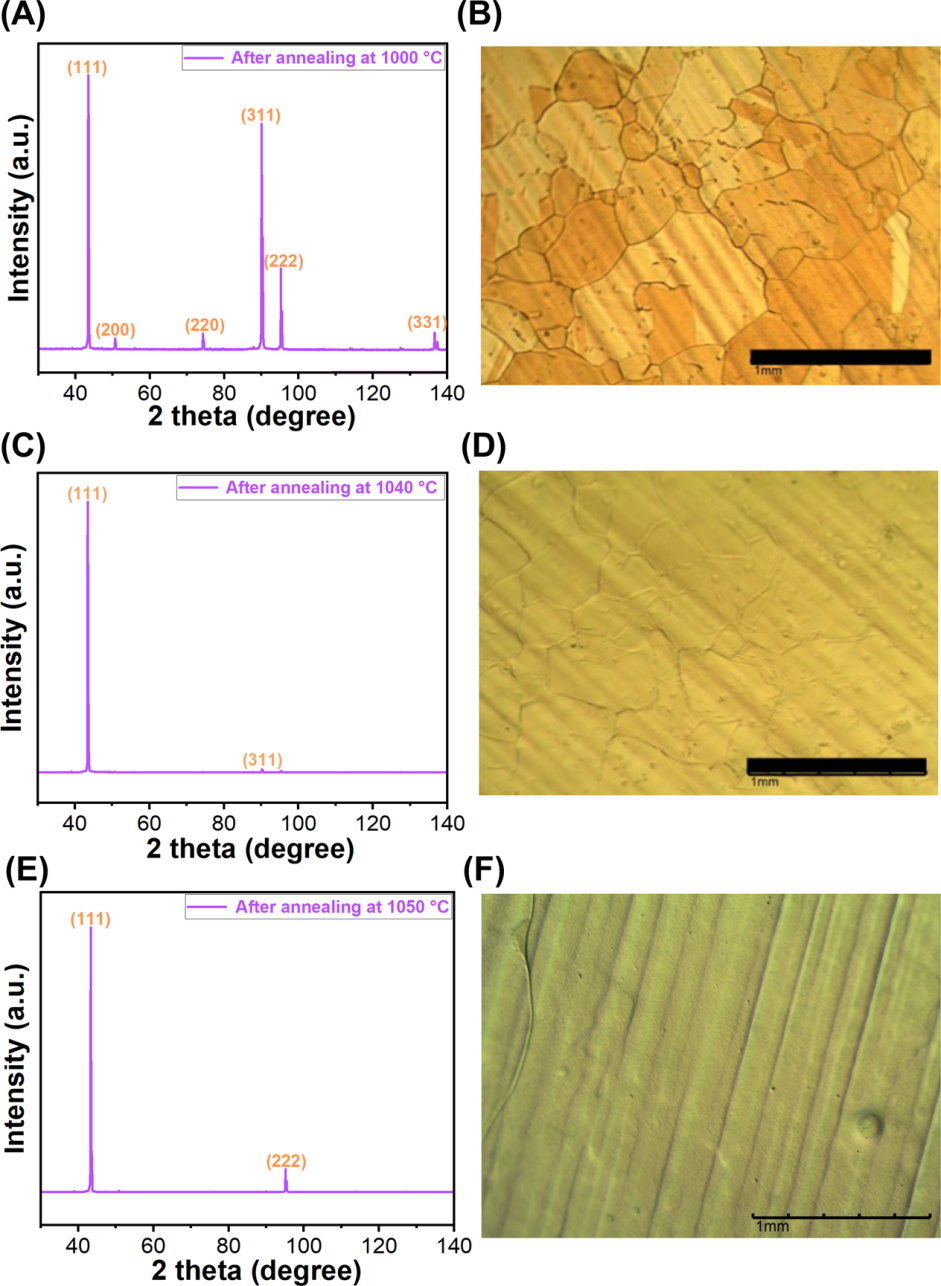
XRD spectra and optical microscopy images of electropolished Cu foils after annealing at (A,B) 1000 °C for 70 min, (C,D) 1040 °C for 3 h, or (E,F) 1050 °C for 3 h. Scale bar: 1 mm.

We investigated the impact of the annealing temperature on the conversion yield and the quality of Cu(111). At 1000 °C for 70 min, the intensity of Cu(111) peak increased and those of (200) and (220) decreased drastically, but the Cu foil remained polycrystalline under this condition (Figure 3A). The optical images revealed heterogeneous grains with sizes ranging from tens of microns to up to 1 mm (Figure 3B). Thermal grooves along the intersects where the grain boundaries meet, which are present in the as-received polycrystalline Cu samples (Figure 1B) and are the result of surface free energy reduction,^30,48^ were still observed on the annealed Cu foil (Figure 3B). After annealing at 1040 °C for 3 h, the XRD spectrum of the Cu foils contained a dominant Cu(111) peak and a weak Cu(311) peak (Figure 3C), and the grain sizes increased (Figure 3D). After annealing at 1050 °C for 3 h, the Cu foil was successfully transformed into single crystalline Cu(111), and other crystalline orientations completely disappeared (Figure 3E). The grain boundaries were significantly reduced, and within the observed image size of 2.5 mm × 2 mm, no complete grain boundary was observed (Figure 3F. The bending line on the left is the grain boundary.^49^). When the annealing temperature reached 1060 °C, no grain boundaries were observed in the 2.5 mm × 2 mm field of view (Figure 2E). In fact, no grain boundaries were observed on a sample of 2 cm × 7 cm in size (Figure S4). These results indicate that annealing at 1050 °C for 3 h was sufficient to transform polycrystalline Cu foil into single crystalline Cu(111). The annealing time of 3 h was shorter compared to, for example, 12–18 h reported by Jin and co-workers and 12 h reported by Brown and co-workers.^30,32^ When the annealing temperature was further increased to 1075 °C, the central area of the Cu foil evaporated (Figure S5) in our low-pressure CVD system (pressure in the system was below 1 Torr). This is likely due to the annealing temperature being very close to the melting point of Cu, 1085 °C.

The impact of annealing time was also studied. Annealing at 1060 °C for 3, 7, or 17 h resulted in the transformation of polycrystalline Cu into Cu(111), as indicated by the presence of only Cu(111) and Cu(222) peaks in the XRD spectra (Figure S6B,D,F). However, the surface of these foils was different. At the annealing time of 3 or 7 h, the Cu foil was smooth and shiny with mirror-like luster (Figure S6A,C). This is due to the removal of dense steps and deep thermal grooves from the Cu surface.^50^ However, when the annealing time was increased to 17 h, the surface became rougher, which can be seen with the naked eye (Figure S6E). This is likely caused by the sublimation of Cu at prolonged annealing time.^51^ Consequently, the quality of CVD graphene grown on Cu(111) prepared under this condition was also affected (see Discussion in the section on graphene growth).

**Figure 4:**
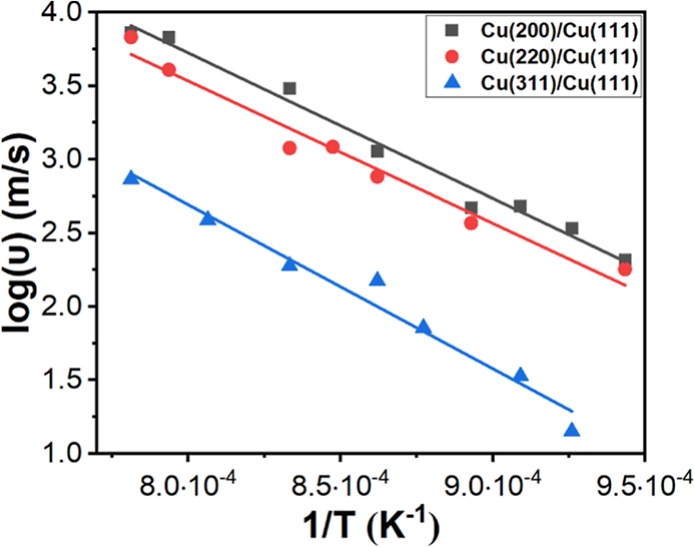
Arrhenius plot of the natural logarithm of the grain boundary migration rate (υ) versus the reciprocal of absolute temperature (1/*T*). Black squares represent Cu(200)/Cu(111) interface, red circles represent Cu(220)/Cu(111) interface, and blue triangles represent Cu(311)/Cu(111) interface.

MD simulations of grain boundary migration were performed to explore the relationship between the grain boundary migration and the annealing temperature. The data collected from MD simulations were presented in an Arrhenius-type plot by linearly expressing the natural logarithm of the approximated grain boundary migration rate (υ) as a function of the reciprocal of absolute temperature (1/*T*) (Figure 4) (see Supporting Information and Figure S7 for the description of simulation models and method). The result shows that the grain boundary migration rate increases with temperature, which is consistent with the experimental observations. Furthermore, at the same temperature, the grain boundary migration rate is the fastest for Cu(200)/Cu(111), followed by Cu(220)/Cu(111), and Cu(311)/Cu(111) is the slowest. Indeed, our experimental data show that while the intensity of Cu(200) is higher than Cu(220) in the Cu foil before annealing (Figure 1H), it is lower than Cu(220) after annealing at 1000 °C for 70 min (Figure 3A). There is still significant amount of Cu(311) after annealing at 1000 °C for 70 min (Figure 3A) and it is the last to disappear (Figure 3C). This could be attributed to its slow grain boundary migration rate (Figure 4) and/or high abundance in the Cu foil (Figure 1H).

### Cu(111) Serves as the Seed for the Conversion of Other Facets to Cu(111)

**Figure 5:**
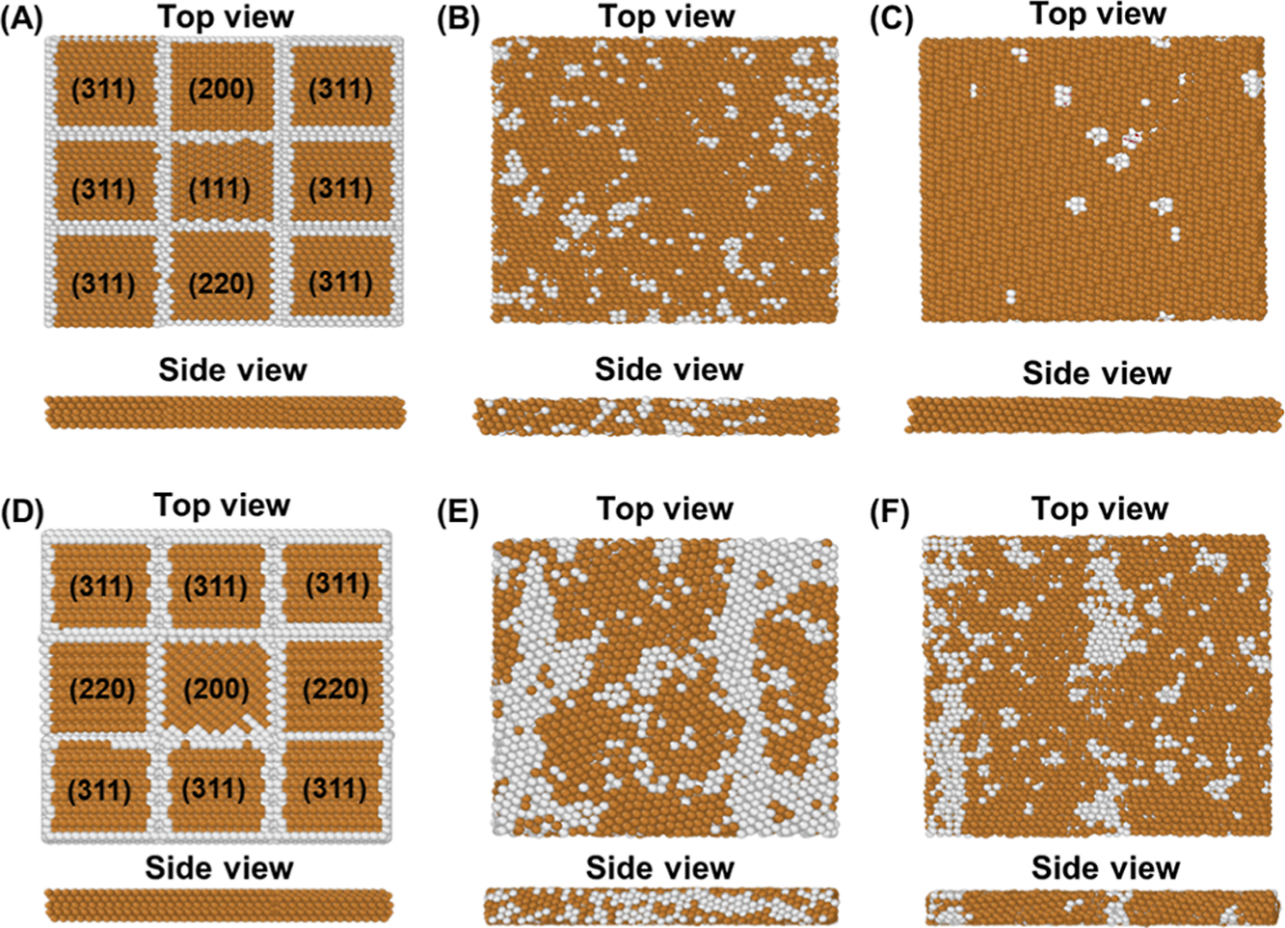
MD simulations of the annealing process: Top and side views of 3 × 3 models (A–C) with and (D–F) without Cu(111) seed block before, during and after annealing. (A) Initial state of the model with Cu(111). (B) State of model with Cu(111) seed block after annealing at 1300 K and 2400 ps. (C) State of model with Cu(111) seed block after annealing at 1300 K for 4800 ps. (D) Initial state of the model without Cu(111) seed block. (E) State of model without Cu(111) seed block after annealing at 1300 K and 2400 ps. (f) State of model without Cu(111) seed block after annealing at 1300 K for 4800 ps. Brown color represents face-centered-cubic (fcc) structure, and white color represents stacking fault displacement. The annealing temperature simulation profile and additional trajectory snapshots are presented in Figures S8 and S9, respectively.

To further explore factors contributing to the successful preparation of Cu(111) foil, MD simulations also performed based on a 3 × 3 model.^34^ Most literature studies used a (200) strong textured Cu foil,^20,33,34,46,50^ while our as-received Cu foils are dominant in (311), (200), (111), and (220) facets (Figure 1D). One hypothesis for the successful conversation of polycrystalline Cu into single-crystal Cu(111) is that the Cu(111) grain serves as the seed for the transformation. To test this, two 3 × 3 Cu models were built: The first has one center Cu(111) block surrounded by six Cu(311) blocks, one Cu(220) and one Cu(200) block (Figure 5A); The second model contains six Cu(311) blocks, two Cu(220) blocks, and one Cu(200) block, but does not have any Cu(111) blocks (Figure 5D). The choice of each crystal plane and their respective quantities reflect the relative intensity in the XRD spectra of as-received Cu foils (Figure 1D). Upon simulating the annealing process, the two models yielded different final structures. The model without the Cu(111) seed shows more white regions representing the fault displacement in the crystal stacking (Figure 5F) than the model with the Cu(111) seed (Figure 5C). It is expected that the white regions would approach zero with a sufficiently long simulation time in the model with the Cu(111) seed. The side views of the two models also show that the model without Cu(111) forms a less uniform crystal orientation compared to the one with a Cu(111) seed. During the annealing simulations, snapshots of trajectories at 2400 ps and 1300 K of both models were taken. Results show that the model without Cu(111) seed contains more crystal stacking dislocations (Figure 5E) compared to that with Cu(111) seed (Figure 5B). These observations support our hypothesis that the presence of the Cu(111) plane plays a significant role in influencing the uniformity of the resulting crystal. This phenomenon could be attributed to the migration and rearrangement of Cu atoms guided by the principle of surface energy minimization, that is, the Cu(111) facet has lower surface energy than other facets, such as (220), (200), and (311).^52^ As a result, the Cu(111) grain serves as seeds, where other facets are converted to (111) under the surface energy minimization principle. Thus, during the annealing process, the grains with higher surface energies migrate or reorient to minimize the overall system energy.^1,2,53^ This transformation is driven by thermal activation and promotes the growth of Cu(111) at the expense of other orientations. Thus, while these planes do not directly support the formation of Cu(111), they contribute indirectly by undergoing conversion during annealing.

### Physical Deformation Significantly Affects Crystal Orientation and Grain Size of Cu Foil

**Figure 6:**
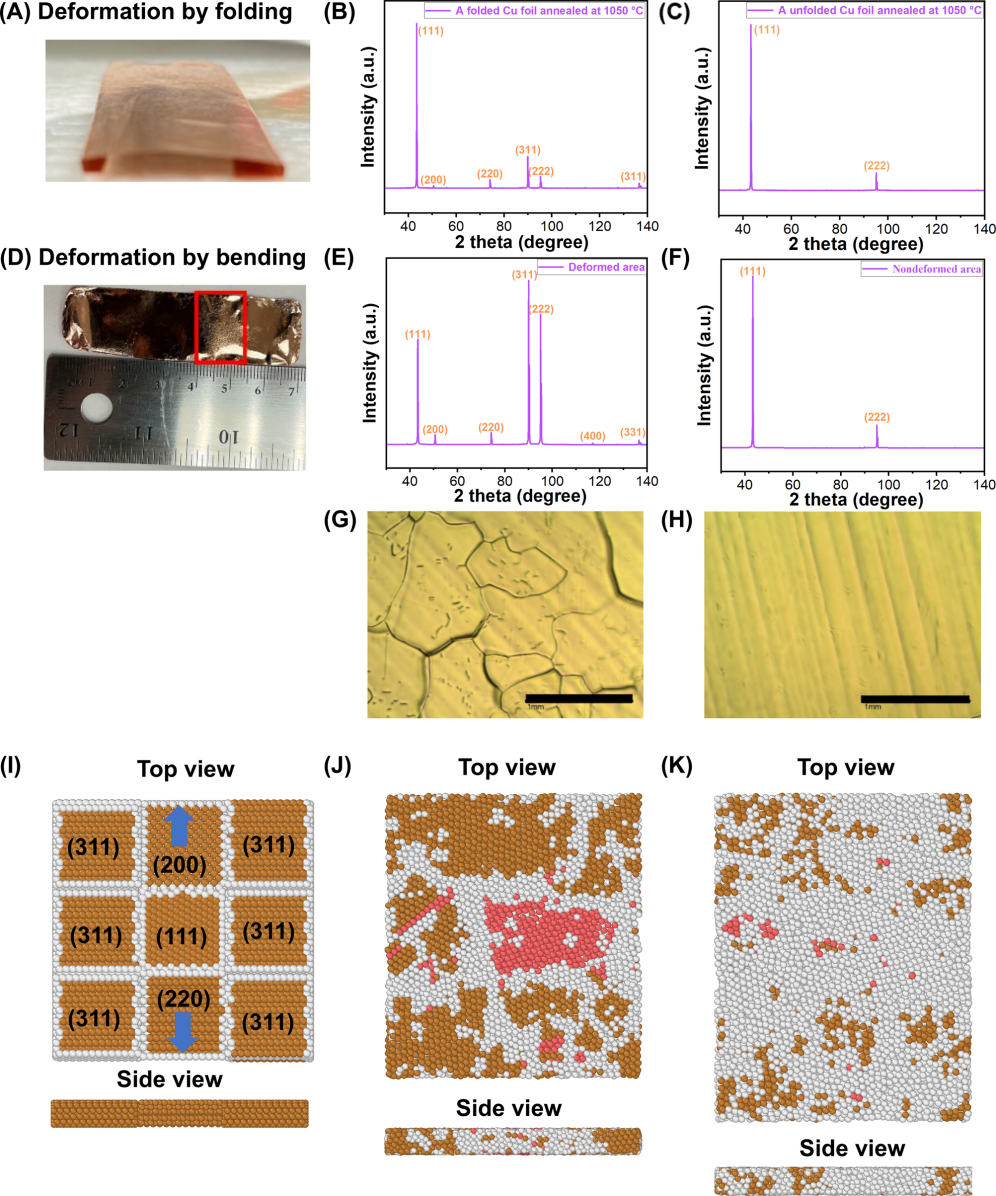
Impact of physical deformation on the grain size and crystal orientation of the annealed Cu foil. (A) Photograph of a folded Cu foil with a “beam bridge” shape prior to annealing. (B) XRD pattern of the folded Cu foil after annealing at 1050 °C for 12 h. (C) XRD spectrum of unfolded Cu foil after annealing at 1050 °C for 12 h. (D) Photograph of a bent Cu foil after annealing at 1060 °C for 3 h. The bent area is marked in the red rectangle. (E) XRD spectrum of the bent area on the Cu foil after annealing at 1060 °C for 3 h. (F) XRD spectrum of the flat area of the annealed Cu foil. (G) Optical microscopy image of the bent area of the annealed Cu foil. (H) Optical microscopy image of the flat area of the annealed Cu foil. (I–K) MD simulation of annealing under uniaxial tension. Top and side views of (I) initial structure of 3 × 3 model, with the direction of uniaxial tensile force shown by the blue arrows, (J) the structure under uniaxial tension at 10 ps, and (K) the structure after completion of MD simulation. The brown color represents fcc structure; the white color represents stacking fault displacement; and the pink color represents hexagonal close-packed (hcp) structure.

Similar to literature reports,^30^ we found that the Cu foil would adhere to the edges of the quartz boat after annealing. We then tested a different configuration where the Cu foil was folded into the “beam bridge” shape and was suspended on a quartz boat (Figure 6A). However, the polycrystalline Cu foil did not completely transform into Cu(111) and multiple crystal orientations were seen after annealing at 1050 °C for 12 h (Figure 6B). This is in contrast to the flat configuration that resulted in the complete transform of polycrystalline Cu to Cu(111) under the same annealing conditions (Figure 6C).

It has been reported that deformed polycrystalline Cu could not be fully converted to single crystalline Cu(111).^30,33,50^ To test whether physical deformation affects the conversion of polycrystalline Cu to Cu(111), we intentionally bent the Cu foil prior to annealing. Indeed, the bent area of the Cu foil (Figure 6D) remained polycrystalline after annealing (Figure 6E), whereas the nondeformed area was completely converted into Cu(111) (Figure 6F). Additionally, in the bent area on the Cu foil, grain boundaries were clearly seen (Figure 6G). On the other hand, the flat regions were free of grain boundaries (Figure 6H) and appeared shinny by the naked eye (Figure 6D).

The incomplete transformation of polycrystalline Cu to Cu(111) could be caused by the strain induced by folding or deformation.^30^ To gain further insights on how the external force-induced deformation inhibits the formation of single crystalline Cu(111), MD simulations were performed by applying a uniaxial tensile force (Figure 6I) during annealing using the 3 × 3 polycrystalline Cu model containing a Cu(111) seed shown in Figure 5A. The annealing led to an increased number of atomic displacements along the trajectories at 10 ps (Figure 6J). In the final snapshot of the simulation, it was clear that much of the area showed displaced stacking faults (Figure 6K). These results underscored the impact of strain induced by uniaxial tension and revealed that strain clearly hindered the formation of a single-oriented Cu(111) plane.

### Purging the CVD System with Working Gases Prevents Cu Foil from Adhering to the Quartz Boat

Initially, we encountered the issue of the Cu foil adhering to the edge of the quartz boat. Additionally, after growing graphene, the Cu foil severely stuck to the quartz boat and the Cu foil also partially evaporated (Figure S10). To test whether this was due to the maximal working temperature of the quartz boat (1200 °C) being close to the annealing temperature (1060 °C), we switched to an alumina boat since alumina has a higher working temperature (1500 °C). However, the Cu foil still stuck to the alumina boat. We then suspected that the air remaining in the gas tube might have entered the furnace when opening the gas gauge. To test this, the valves of the three gas gauges, including hydrogen, methane, and argon, were opened to purge the CVD chamber prior to annealing. This simple operation proved to be critical: The annealed Cu foils no longer stuck to the quartz boat. The Cu foil remained intact without any breakage or evaporation and the surface of the annealed Cu(111) foils was smooth and shiny (Figure 2C). It has been reported that in the presence of oxygen, Cu can be oxidized at high temperatures to produce Cu–O–Si_*x*_ or other oxides and silicides at the Cu/quartz (SiO_2_) interface.^54^ We suspect that the residual air (oxygen) in the CVD chamber promoted the covalent bond formation between Cu and the quartz during high temperature annealing, causing the adherence of Cu foil to the quartz boat. Purging the system with working gases cleared the residual air and prevented the oxidative reaction of Cu with the quartz boat.

### Fabrication of Monolayer Graphene on Cu(111)

The growth of graphene can be achieved directly on Cu(111) foils without opening the CVD chamber or the need of additional operations. This was carried out by closing the argon gas valve and opening the methane gas valve. This not only simplifies the fabrication process, but also avoids oxidation of Cu(111) if it is exposed to air. To grow graphene, methane (3–6 sccm) was introduced into the quartz tube at the same temperature of 1060 °C for 30 min. Afterward, the furnace was cooled rapidly under hydrogen and methane. The temperature profile of the furnace during graphene growth is shown in Figure S11. The flow rate of methane was kept below 10 sccm during growth. We observed that higher methane flow rates yielded multilayer graphene.

Graphene fabricated on Cu(111) were characterized by AFM, optical and SEM microscopy, and XRD. The RMS, 18.8 ± 6.0 nm (Figures 2H, and S1J–L), is slightly lower than the RMS of Cu(111) at 21.0 ± 4.2 nm (Figure 2D). Both optical and SEM images show uniform and grain boundary-free morphology (Figure 2I,J). The graphene layer on top of Cu(111) not only reduced the surface roughness, but also masked the striations. The grooves on Cu(111) (Figure 2E) was much less obvious after graphene growth (Figure 2I). The XRD spectrum of Cu(111) covered with graphene was similar to that of Cu(111), showing only the (111) and (222) facets (Figure 2K).

Raman spectroscopy is a work horse for the characterization of graphene, including the number of layers, the presence of physical defects, and the extent of chemical functionalization.^55^ To characterize graphene by Raman spectroscopy, graphene fabricated on Cu(111) foil was first transferred to a silicon wafer having a 280 nm oxide layer, as it provides maximal Raman signal enhancement for graphene.^56^ We first optimized the Raman laser power and irradiation time. It has been reported that prolonged laser irradiation can cause oxidation and/or structural damage to graphene, the extent of which can be determined by measuring the intensity of the D peak versus that of the G peak, i.e., *I*_D_/*I*_G_.^57^ On the other hand, low laser power will give spectra having high spectral noises. To determine the optimal conditions, Raman spectra were collected at laser power of 0.2 mW or 1 mW for 1, 5, or 10 s, respectively. The results and detailed discussions can be found in Supporting Information and Figure S12. From these studies, the laser power of 1 mW and irradiation time of 5 or 10 s were used for the subsequent Raman characterization unless otherwise noted.

### Impact of Electropolishing and Annealing Conditions of Cu Foil on the Quality of Graphene

Graphene was fabricated with or without electropolishing and under different annealing temperatures and durations. The samples were examined under optical microscopy and subjected to Raman characterization (Figure S13). The Raman full width at half-maximum of the G (FWHM_G_) and 2D peak (FWHM_2D_), *I*_2D_/*I*_G_, and *I*_D_/*I*_G_ are summarized in Table 1. For graphene grown on as-received polycrystalline Cu foils (entry 1), the D peak can be seen in some but not all areas (Figure S13A), and the *I*_D_/*I*_G_ was 0.081 ± 0.059 (Figure S13C). The graphene was uneven and broken pieces were visible, likely caused by the rough Cu surface (Figure S13B).^58^ Graphene grown on electropolished Cu (entry 2) was more uniform (Figure S13E) than those grown on unpolished Cu (Figure S13B), however, the D peak was still visible in the Raman spectrum (Figure S13D), with the *I*_D_/*I*_G_ of 0.207 ± 0.138 (Figure S13F). Under the annealing condition of 1000 °C for 70 min, the Cu foil was polycrystalline (cf. Figure 3A) with many grain boundaries (Figure 3B). These could contribute to a higher number of physical defects in graphene and therefore larger *I*_D_/*I*_G_. The quality of graphene grown on Cu foils annealed at 1040 °C (entry 3) or 1050 °C for 3 h (entry 4) showed obvious improvement, with *I*_D_/*I*_G_ decreasing to 0.076 ± 0.035 and 0.125 ± 0.049, respectively (Figure S13H,J). This is consistent with decreasing grain boundary and increasing grain size of Cu foils annealed under these conditions (Figure 3D,F). Graphene grown on Cu foils annealed at 1060 °C for 2 h was of relative good quality, with *I*_D_/*I*_G_ of 0.092 ± 0.043 (entry 5, Figure S13K,L). Graphene grown on Cu foils annealed at 1060 °C for 3 h gave the lowest *I*_D_/*I*_G_ of 0.048 ± 0.026 (entry 6). However, when graphene was grown on Cu foils annealed at 1060 °C for 17 h, *I*_D_/*I*_G_ increased to 0.314 ± 0.098 (entry 7, Figure S13M,N). The significant deterioration in the quality of graphene is closely correlated with the increased roughness of Cu foil: The Cu foil annealed at 1060 °C for 17 h had a much rougher surface due to evaporation of Cu (Figure S6E).

**Table 1:** Impact of Electropolishing and Annealing Conditions of Polycrystalline Cu Foils on the Raman Characteristics of Graphene^a^

entry	electropolishing	annealing conditions	FWHM_G_	FWHM_2D_	*I*_2D_/*I*_G_	*I*_D_/*I*_G_
1	no	1000 °C, 1 h	21.7 ± 2.7	35.1 ± 3.3	1.94 ± 0.67	0.081 ± 0.059
2	yes	1000 °C, 70 min	20.4 ± 1.8	31.3 ± 2.0	2.81 ± 0.84	0.207 ± 0.138
3	yes	1040 °C, 3 h	22.2 ± 0.8	35.3 ± 0.8	2.18 ± 0.25	0.076 ± 0.035
4	yes	1050 °C, 3 h	23.6 ± 1.8	37.0 ± 2.7	2.32 ± 0.62	0.125 ± 0.049
5	yes	1060 °C, 2 h	24.1 ± 1.9	36.1 ± 2.5	3.00 ± 0.30	0.092 ± 0.043
6	yes	1060 °C, 3 h	23.7 ± 1.4	38.7 ± 2.9	2.54 ± 0.71	0.048 ± 0.026
7	yes	1060 °C, 17 h	25.4 ± 1.5	38.4 ± 1.6	2.42 ± 0.49	0.314 ± 0.098

a Results were the average ±SD (standard deviation) of data from multiple spectra, collected at random locations on two different samples. The Raman spectra, FWHM_G_, FWHM_2D_, *I*_2D_/*I*_G_, and *I*_D_/*I*_G_ can be found in Figure S13.

**Figure 7:**
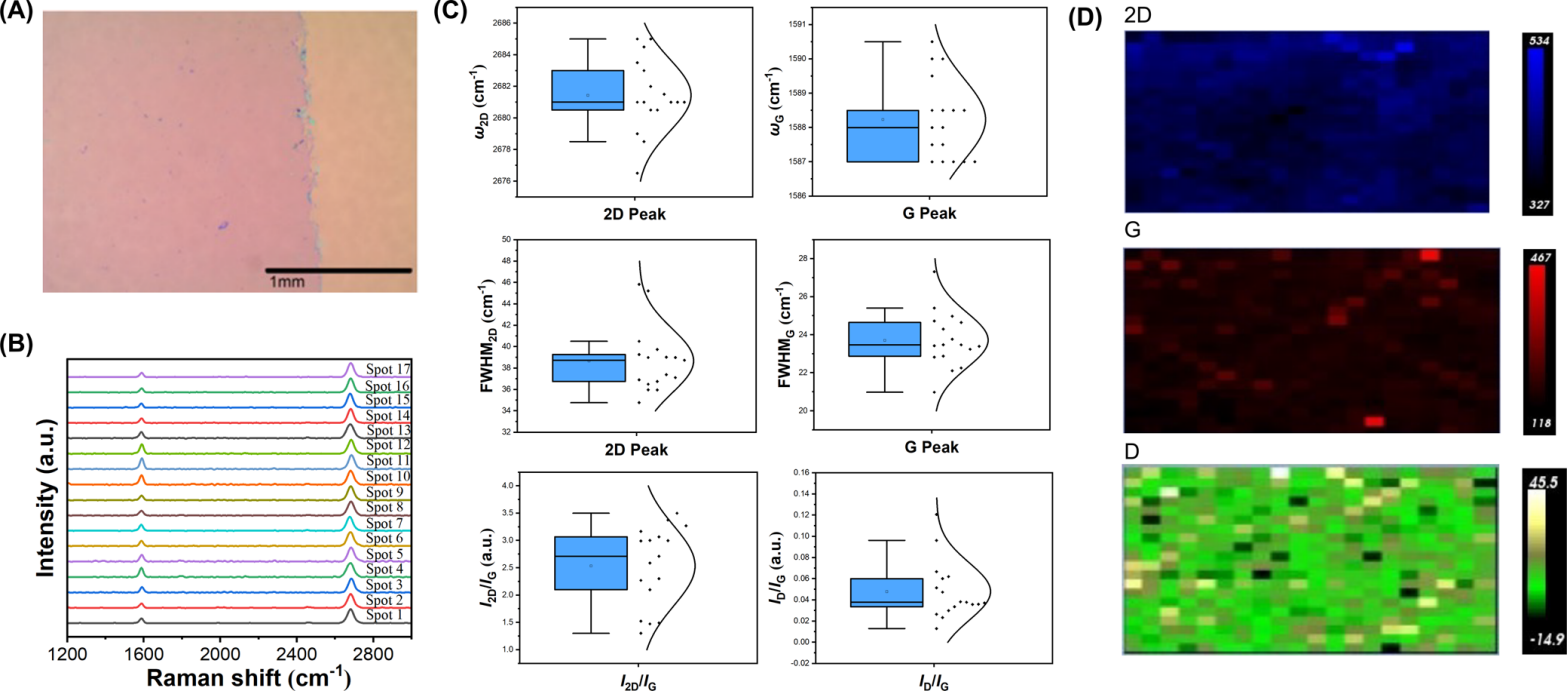
Characterization of graphene grown on Cu(111) fabricated by annealing electropolished Cu foil at 1060 °C for 3 h. (A) Optical microscopy image of graphene after transferring to a silicon wafer. The pink area is graphene, and the yellow area is the silicon wafer. The purple dots are likely due to the incomplete removal of poly(methyl methacrylate) (PMMA) used during graphene transfer.^37^ We have shown that the residual PMMA does not alter the Raman characteristics of graphene.^24,25^ (B) Raman spectra collected at 17 different locations on graphene randomly selected from two samples. Each sample was about 0.5 cm × 0.5 cm in size. Spectra were collected using the laser power of 1 mW and irradiation time of 5 s. An in-house Python code was used to analyze all spectra, with the baseline corrected to obtain accurate peak intensity.^36^ (C) Boxplots of all Raman spectroscopy data in B: 2D peak position, G peak position, FWHM_G_, FWHM_2D_, *I*_2D_/*I*_G,_ and *I*_D_/*I*_G_. The box represents the average; the horizontal black line in the box is the median, and the curve on the right side of each figure is the statistical distribution of the data. The data values are listed in Table S2. (D) Raman mapping of 2D, G and D peaks intensity of graphene.

The highest quality graphene was obtained on Cu foils that were electropolished and annealed at 1060 °C for 3 h (entry 6). The optical microscopy image showed a uniform and continuous graphene layer after it was transferred to a silicon wafer (Figure 7A). In all spectra, the D peak at ∼1350 cm^–1^ are mostly absent (Figure 7B). Boxplots revealed the average 2D and G peak values at 2687.0 and 1590.6 cm^–1^, respectively. FWHM_2D_ and FWHM_G_ were 38.7 ± 2.9 cm^–1^ and 23.7 ± 1.4 cm^–1^, respectively. The average *I*_2D_/*I*_G_ was greater than 2, which is indicative of single-layer graphene.^59^ The *I*_D_/*I*_G_ was 0.048 ± 0.026. These results are consistent with high-quality monolayer graphene grown on Cu(111).^12,16,28,50^ The prepared graphene was further characterized by Raman mapping over a 30  μm × 30  μm area with a step size of 1.5 μm, giving a total of data 400 points (Figure 7D). The calculated *I*_2D_/*I*_G_ and *I*_D_/*I*_G_ were 2.56 ± 0.43 and 0.050 ± 0.070, respectively. These values align closely with the measurements obtained from Raman spectra collected randomly across the entire samples, indicating uniformity in graphene quality. In addition, the method is reproducible, giving graphene of consistent quality (see Figures S14 and S15 and Tables S3 and S4 for the characterization of two additional batches).

## Discussion

The tube furnace is a relatively low-cost equipment commonly used in academic research laboratories. We built a CVD setup from the Lindberg/Blue M mini-mite tube furnace (Figure S16). The furnace accommodates a 1″-diameter quartz tube, which allows us to fabricate graphene on a 7 cm × 2 cm Cu foil. The fabrication of Cu(111) from polycrystalline Cu foils consists of two main steps: electropolishing followed by annealing. The commercial polycrystalline Cu foils are rough, showing obvious striations (Figure 1A–C). The nucleation density along the striations is high due to the low mobility of carbon-adatom species.^60^ This leads to relatively high nucleation and high density of graphene grain boundaries in the striation area than in the flat region of the Cu foil. Electropolishing offers a convenient way to reduce striations and smooth the surface of Cu foils. It has been reported that CVD graphene grown on electropolished Cu foils had fewer grain boundaries and larger grain sizes.^43,44^ In our case, the RMS roughness of polycrystalline Cu foils decreased from 113 ± 12 nm to 38.8 ± 12 nm after electropolishing at the current of 2.80 A for 2.5 min. The density of striations also decreased. After electropolishing, the intensity of the (200) and (220) facets relative to that of (311) decreased from 94% and 59% (Figure 1D) to 37% and 12%, respectively (Figure 1H).

In the strain-free method for the fabrication of Cu(111) developed by Ruoff and co-workers, a custom-made quartz holder was used to secure the Cu foil and reduce the strain. To accommodate the quartz holder, a larger quartz tube was necessary, and thus a more expensive furnace. In our setup, the Cu foil is placed horizontally on top of the quartz boat, which is commonly used in the fabrication of CVD graphene (Figure 2). Several factors are crucial for achieving the conversion of polycrystalline Cu to single crystal Cu(111): (1) deformation-free substrate. Only polycrystalline Cu foils without physical deformation can be fully converted to single crystalline Cu(111). Bending or distorting the Cu foil prevented the complete conversion of polycrystalline Cu to single crystal Cu(111) (Figure 6), a result which is also supported by MD simulations (Figure 6I–K). (2) Air-free environment. We initially encounted the issue of Cu foil adhering to the quartz boat. This issue was completely eliminated after the CVD system was purged with the working gases prior to annealing. We suspected that in air and at high temperature (>600 °C), Cu undergoes oxidative reaction with quartz (SiO_2_), which bonds Cu to the quartz boat. (3) Annealing temperature and time. The annealing temperature turned out to be the most critical parameter. We found annealing at 1060 °C for 3 h to be optimal to convert polycrystalline Cu to single crystal Cu(111) using the configuration shown in Figure 2A. Annealing at temperatures lower than 1060 °C for 3 h either did not completely convert polycrystalline Cu to Cu(111) (Figure 3A–D), or give smaller Cu(111) grains and rougher surface (Figure 3E,F). The impact of annealing temperature on the conversion of polycrystalline Cu to Cu(111) was also supported by an Arrhenius analysis from MD simulations (Figure 4). Higher annealing temperature promotes grain boundary migration, and the migration rate from facets (200), (220), or (311) to (111) increases with the annealing temperature. Furthermore, it has been reported that high temperature near the melting point of Cu (1085 °C) promotes vacancy formation and diffusion.^49^ This enables crystal lattice rotation and the removal of defects such as stacking faults, which are crucial for the transformation of polycrystalline into single-crystal structure.

The MD simulations in Figure 5 show a discernible difference in simulation results with and without Cu(111) seed grains. A better uniformity was obtained for the model with Cu(111) seed grain after annealing. Specifically, the data from Figure 5C compared to Figure 5F underscores the role of Cu(111) seed grains in facilitating the formation of single crystal Cu(111) structure. The dominant orientations of as-received Cu foils are Cu(311), Cu(200), Cu(111), and Cu(220) (Figure 1D). Compared to Cu(111), other crystal orientations, such as Cu(311), Cu(200), and Cu(220), have higher surface energies.^61^ During the annealing process, the grains with higher surface energies migrate or reorient to minimize the overall energy.^1,2,53^ This transformation is driven by energy minimization, which promotes the growth of Cu(111) at the expense of other orientations.

We acknowledge that as the size of the Cu foil increases, the risk of Cu foil deformation and evaporation could potentially become more significant due to gravitational effects from the weight of the foil itself. Scaling up production would likely require optimization to support the Cu foil and prevent deformation, for example, by using reinforced supports or alternative boat design. Another approach is to lower the annealing temperature. This would increase the annealing time as the grain boundary migration rate will decrease at lower annealing temperature, as shown in the Arrhenius plot in Figure 4.

The setup in Figure 2A allows us to grow graphene directly on the annealed Cu(111) foil without opening the CVD chamber or introducing any additional apparatus. This not only simplifies the fabrication process, but also avoids oxidation of Cu(111) when it is exposed to air. Additionally, it eliminates contaminants that could interfere with graphene growth or increase nucleation sites which can prevent the growth of large-area graphene.^62^ To grow graphene, the flow rate of methane was set at below 10 sccm for 30 min. We observed that higher methane flow rates yielded multilayer graphene. At high temperatures, low methane flow rate and partial pressure lead to low density of graphene nuclei, allowing individual graphene domains to grow larger before coalescing. This condition is favored for generating high quality graphene with larger domain sizes due to fewer interdomain defects.^63^ On the other hand, high methane flow rate and partial pressure result in high density of graphene nuclei, which leads to smaller domains as they quickly coalesce.

The best quality graphene was obtained on Cu foils annealed at 1060 °C for 3 h. This is not surprising as this condition gave large single crystalline Cu(111) having smooth surface. In addition, Cu foil annealed at 1060 °C for 3 h gave the lowest amount of grain boundaries. This reduces the density of graphene nucleation sites, resulting in larger grains of high quality graphene.^13,64^ Annealing at lower temperatures produced graphene having obvious D peak, likely resulting from the physical defects caused by small grains and high number of grain boundaries. Longer annealing time of 17 h at 1060 °C produced graphene with more defects. This can be attributed to the Cu foils having rough and damaged surface due to Cu evaporation under these conditions, which led to higher nucleation density and more graphene grain boundaries. Taken together, large grains of single crystalline Cu(111) having a smooth surface are critical in obtaining higher quality monolayer graphene with no or minimal defects.

It has been widely reported that Cu(111) is well-suited for the epitaxial growth of graphene due to its *C*_3_ rotational symmetry that closely matches graphene hexagonal structure and thus promoting a preferred orientation of graphene with minimal rotational misalignment between the graphene lattice and the Cu(111) substrate.^13^ Besides, graphene grown on Cu(111) exhibits excellent quality due to small lattice mismatch (approximately 3–4%) between Cu(111) and graphene, which further promotes the growth of graphene with a single orientation, ensuring high-quality monolayer graphene with minimal defects.^12^ High index copper has also been used to grow high-quality graphene with single orientation.^65,66^ However, on high-index facets of Cu, the graphene orientation is less predictable and exhibits more variation in alignment due to the complex atomic arrangements of these facets.^67^ High-index copper foils contain steps, edges and kinks with special symmetries, making it challenging to control the quality of graphene.^68,69^

## Conclusions

In conclusion, we have developed a streamlined process to convert polycrystalline Cu to Cu(111) and subsequently grow high quality monolayer graphene with no or minimal defects. Cu(111) was obtained by annealing the commercially available and inexpensive polycrystalline Cu foils at 1060 °C for 3 h. No special holder or additional apparatus was needed, and the annealing was achieved by placing the polycrystalline Cu foil on a quartz boat in a tube furnace that is commonly used in academic research laboratories. We have conducted systematic studies to investigate the impact of electropolishing and annealing conditions on the conversion of polycrystalline Cu to Cu(111). Our results showed that the annealing temperature is the most critical factor in producing large areas of single crystalline Cu(111). Annealing polycrystalline Cu at 1060 °C for 3 h led to the conversion to Cu(111) across the sample area of 7 cm × 2 cm.

Additionally, we discovered that a common issue in CVD fabrication, which is Cu foil adhering to the quartz boat, was due to the residual air in the system and can be resolved by purging the system with the working gases prior to annealing. This simple operation also prevented the evaporation of Cu foil which occurred without implementing this procedure.

We have shown that graphene can be grown directly on the annealed Cu foil simply by introducing the precursor gases into the CVD chamber. We investigated the impact of substrate preparation and annealing conditions on the quality of graphene, and found that smooth surface and large grain size of Cu(111) were critical in obtaining high quality graphene. As it takes 3 h to prepare Cu(111), 30 min to grow graphene, and 1 h 40 min to cool the system down to room temperature, the entire process can be completed in the home-built CVD in about 5 h without extra steps or using additional apparatus. This simplified procedure allows for cost-effective synthesis of high-quality graphene to be readily accessible to academic researchers for a wide range of applications, including nanoelectronics.^3^

## Supporting Information Available

Comparison of literature and this method on Cu(111) fabrication conditions; electropolishing setup and protocol; additional AFM and optical images of Cu foils treated under different conditions; additional MD simulation results and temperature profile; optimization of laser power and irradiation time for Raman spectroscopy; optimization of annealing temperature for graphene growth; additional Raman data and optical images of graphene; CVD setup and temperature profile of the furnace (PDF)
