# Supplementary material for: Chemical Vapor Deposition of Monolayer Graphene on Centimeter-Sized Cu(111) for Nanoelectronics Applications
Source: ACS Appl Nano Mater. 2025 Feb 24;8(9):4926–39. doi: 10.1021/acsanm.5c00588 (PMC11894592; doi:10.1021/acsanm.5c00588)
Supplement: Supplementary file 1 — an5c00588_si_001.pdf [file an5c00588_si_001.pdf]

Supporting Information

Chemical Vapor Deposition of Monolayer Graphene on Centimeter-Sized Cu(111) for Nanoelectronics Applications

Jia Tu, Wentong Zhou, Amin Kiani, Lawrence M. Wolf,* Mingdi Yan*
Department of Chemistry, University of Massachusetts Lowell, Lowell, MA 01854, United States
* Corresponding authors: Mingdi_Yan@uml.edu, Lawrence M. Wolf@uml.edu

Table of Contents

1. Summary of different conditions for the fabrication of Cu(111)....S2

2. AFM images of polycrystalline Cu foils before and after electropolishing and annealing, and graphene grown on Cu(111)....S3

3. Electropolishing setup....S4

4. Impact of electropolishing current on the morphology of electropolished Cu foils....S4

5. Optical microscopy images of Cu(111) foils....S5

6. Cu foil annealed at 1075 °C....S5

7. Impact of annealing time on the quality of Cu(111)....S6

8. MD simulation of grain boundary migration at variable temperature....S6

9. Temperature profile of MD simulation and additional trajectory snapshots. S8

10. Annealing Cu foils in the presence of residual air in the CVD chamber....S9

11. Temperature profile of the furnace during graphene growth....S9

12. Optimization of laser power and irradiation time for Raman spectroscopy....S9

13. Optimization of annealing conditions for the growth of monolayer graphene....S11

14. Summary of the Raman data and characterization of two additional batches of graphene grown on electropolished Cu foil annealed at 1060 °C for 3 h. S13

15. Home-built CVD setup....S16

16. Additional two batches of graphene on Cu(111)....S17

References....S18

1. Summary of different conditions for the fabrication of Cu(111)

Table S1. Fabrication conditions for Cu(111) via abnormal grain growth: Comparison of literature and this method

Cu Foil Source	Atmosphere	Apparatus	Annealing Temperature	Annealing Time	Product Purity	Size of Cu(111)	Note	Reference
Nilaco #CU-113213, 99.9%	Hydrogen (100 sccm) and argon at a total pressure of 26 Torr.	Hot-wall quartz tube furnace	1030 °C	Up to 12 h	~95% Cu(111)	Millimeter		[1]
Nilaco, 100 μm thick, 99.96%	Ar (1000 sccm) and H_2 (500 sccm)	Atmospheric CVD	1075 °C	> 4 h	98% Cu(111)	Millimeter		[2]
Alfa Aesar, 25 μm thick, 99.8%	Ar (2000 sccm) for 15 min with residual oxygen, then 500 sccm, with the delayed addition of 20 sccm of hydrogen	Hotwall furnace	1050 °C		N/A	Millimeter	With oxyge[3]n	[3]
Nilaco Co., 80 μm thick, 99.9%	Hydrogen and argon, both at 10 sccm and a pressure of 760 Torr	Atmospheric CVD	1050 °C	12-18 h	N/A	Centimeter	Without strain	[4, 5]
35 μm thick, 99.7%	Hydrogen (50 sccm) at 970 °C for 1 h at 3.8×10^{-1} Torr	Vertical CVD	970 °C	1 h	N/A	Millimeter	With strain	[6]
Sichuan Oriental Stars Trading Co. Ltd., 25 μm thick, 99.8%	500 sccm Ar	CVD furnace with roll-to-roll setup	1030 °C	2.5 cm/min in 50 min	N/A	Meter		[7]
N/A	Heat under Ar (1000 Pa), and then hydrogen (1000 Pa) after maintaining the temperature gradient for 20 min.	Homemade low-pressure CVD system equipped with a 6” quartz tube, or a self-designed industrial CVD system	Temperature gradient from 1040 to 920 °C (about 2 °C/cm along the axial direction) and then minimization of temperature gradient	N/A	N/A	Centimeter	Tempera ture gradient	[8]
Kunshan luzhifa Electronic Technology Co., 25 μm thick	Cu foil was heated at 400-450 °C for 30 min to oxidize the surface. After reducing the pressure to 8 Pa, the Cu foil was heated to 1040 °C at 20 °C/min in the presence of 1000 sccm H_2 (300 Pa), and then annealed at 1040 °C in the same atmosphere for 60 min.	Home-built CVD system (Lindberg/Blue M Tube Furnace, HTF55667C)	1040 °C	More than 2 h (including oxidation time)	N/A	Meter	With oxygen	[9]
Alfa Aesar, 0.025 mm thick, annealed, uncoated, 99.8%	Argon (15 mTorr) and Torr H_2 (10 mTorr) at the pressure of about 1000 mTorr	Home-built CVD setup (Lindberg/Blue mini-mite Tube Furnace, TF55030 A-1)	1060 °C	3 h	100%	Centimeter	Without oxygen or strain	This work

2. AFM images of polycrystalline Cu foils before and after electropolishing and annealing, and graphene grown on Cu(111)

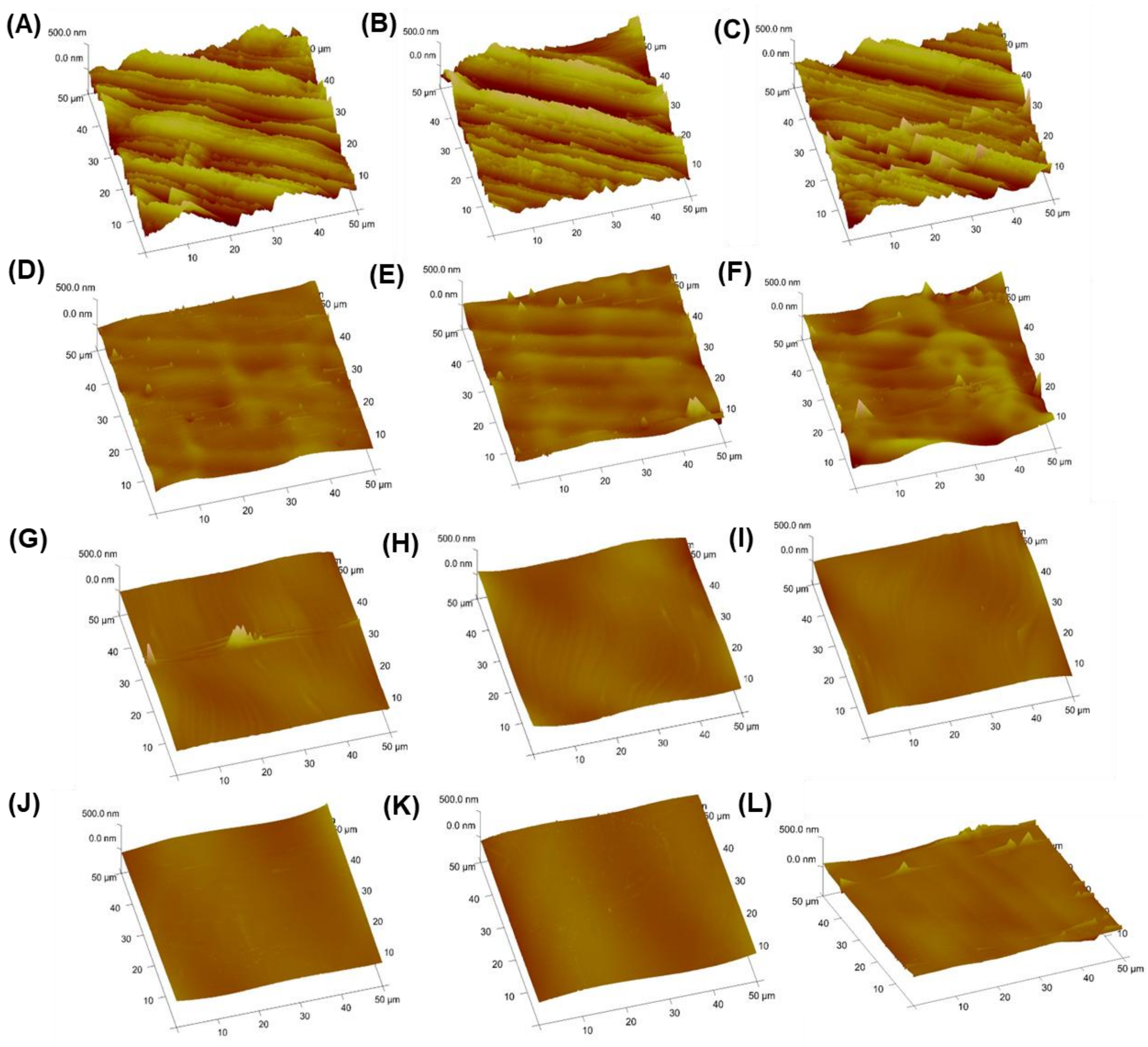

Figure S1. AFM images of (A, B, C) as-received polycrystalline Cu foil, (D, E, F) polycrystalline Cu foil after electropolishing, (G, H, I) Cu(111) foil after annealing at 1060 °C for 3 h, and (J, K, L) graphene grown on Cu(111).

3. Electropolishing setup

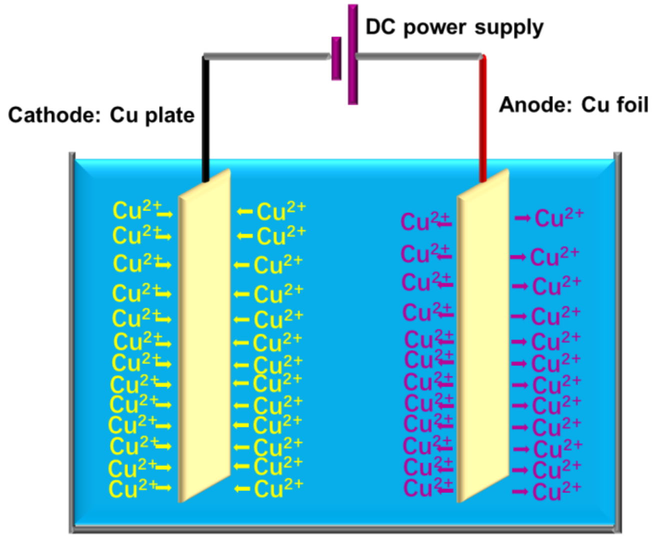

Figure S2. The electropolishing setup, consisting of a TekPower DC regulated power supply (TP-3003D), connecting cables (Item #20FP83, Grainger), a Cu plate (0.675 mm thick, annealed, 99.9%, Alfa Aesar) as the cathode, and the Cu foil (0.025 mm thick, annealed, uncoated, 99.8%, Alfa Aesar) as the anode. The anode and the cathode were attached to the connecting cable using metal alligator clips. The electropolishing solution was prepared from 1000 mL of Milli-Q water, 500 mL of phosphoric acid, 500 mL of ethanol, 100 mL of isopropyl alcohol, and 10.0 g of urea.

4. Impact of electropolishing current on the morphology of electropolished Cu foils

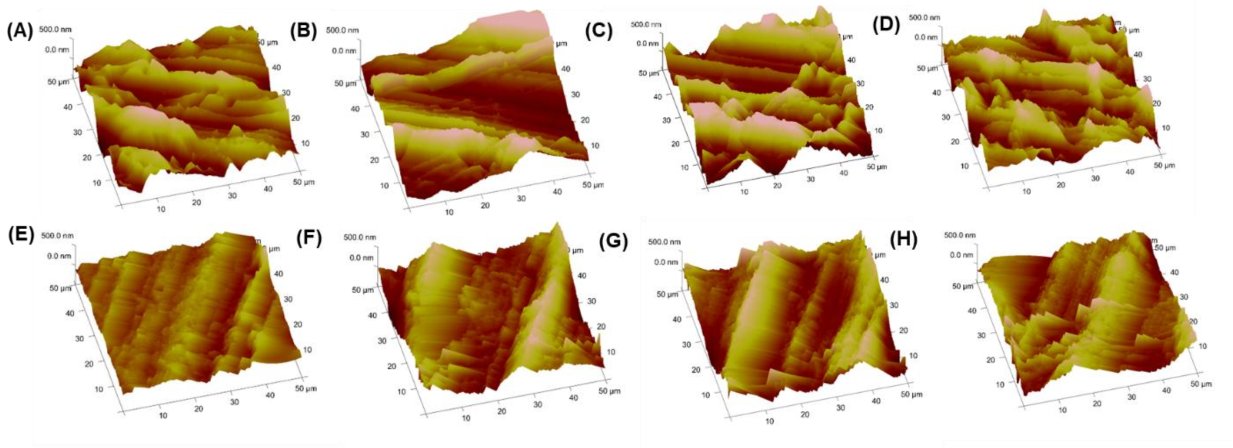

Figure S3. AFM images of polycrystalline Cu foil after electropolishing at the current of (A–D) 0.70 A, or (E–H) 1.70 A for 2.5 min.

5. Optical microscopy images of Cu(111) foils

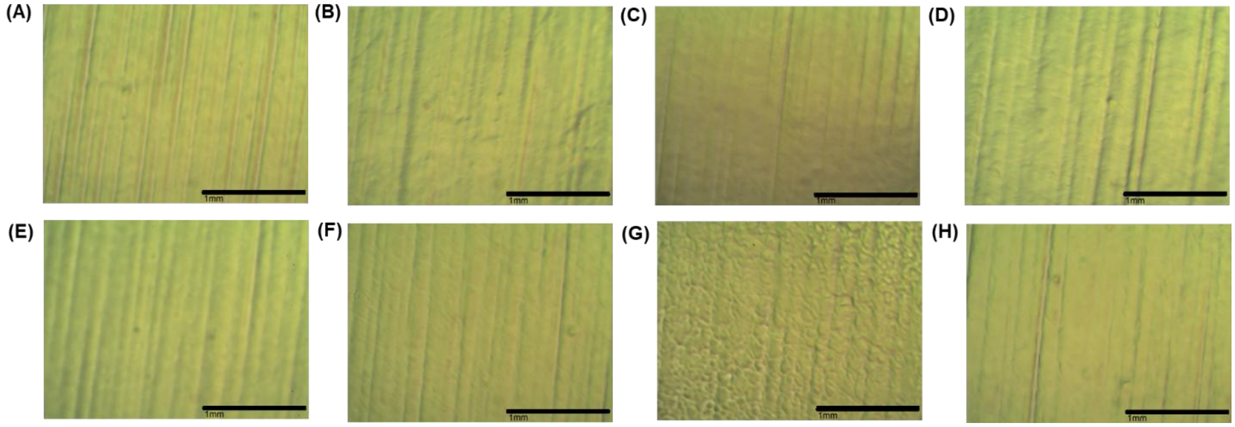

Figure S4. Optical microscopy images of annealed Cu(111) foil at different locations on the sample of 2 cm × 7 cm. Scale bars: 1 mm.

6. Cu foil annealed at 1075 °C

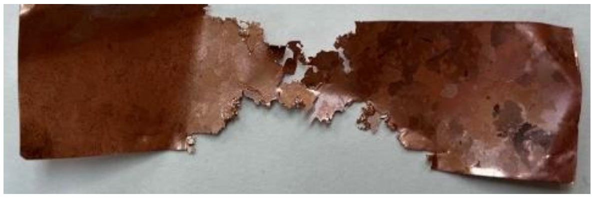

Figure S5. Photograph of electropolished polycrystalline Cu foil after annealing at 1075 °C for about 3 h.

7. Impact of annealing time on the quality of Cu(111)

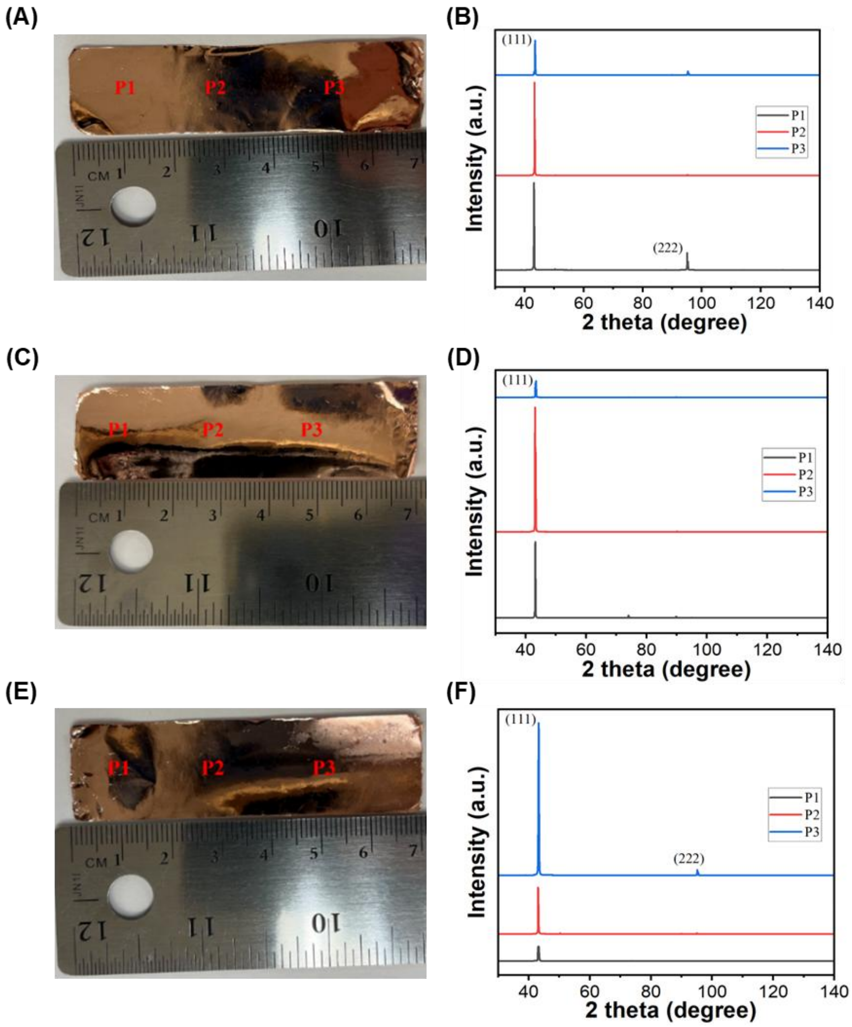

Figure S6. Photographs and XRD spectra of Cu foils annealed at 1060 °C for (A, B) 3 h, (C, D) 7 h, and (E, F) 17 h. After annealing, each 2 cm × 7 cm Cu foil was cut into three pieces (P1, P2, P3) at around 2 cm × 2 cm each, and the XRD spectra of all three pieces were collected.

8. MD simulation of grain boundary migration at variable temperature

Migration of grain boundary interface is thermally activated and follows an Arrhenius relation:

$$M^* = M_0 e^{-\frac{Q}{kT}}$$

Where M^* is reduced grain boundary mobility, M_0 is the pre-exponential factor, Q is the activation energy barrier for grain boundary migration, k is the Boltzmann constant, and T is the absolute temperature.

The relation between grain boundary migration rate (v) and grain boundary mobility (M) is as below, in which F represents the driving force of grain boundary migration:

$$v = MF$$

The models we built for variable-temperature grain boundary migration simulations are two same-size single crystal plane Cu blocks along the x-axis, and the interface between the two parts is along the y-axis, shown in **Fig. S7**.

In our simplified two-crystal-plane-interface model, the migration would be along the x-axis, so the driving force (*F*) could be simplified as the ratio between grain boundary energy (γ) and length of the single-crystal plane block Cu along the x-axis (l)

$$F = \frac{\gamma}{l}$$

The reduced grain boundary mobility is shown as $M^* = \gamma M$. By combining the two equations above, we could get the reduced grain boundary mobility along the x-axis as below:

$$M^* = l\frac{dl}{dt}$$

Then the Arrhenius plot could be made between the natural logarithm of M^* and the reciprocal of temperature ($1/T$). Since the time scale of our simulation is short, the length (l) could be seen as a constant number, so we directly plot the natural logarithm of grain boundary migration rate (v) versus the reciprocal of temperature ($1/T$).

The grain boundary migration was approximated by measuring the maximal coordinates change along the x-axis before and after the MD simulation, which was then divided by the total time. To obtain statistically meaningful results, average values were obtained after MD simulations at each specific temperature and each model were run ten times with random initial velocity seeds.

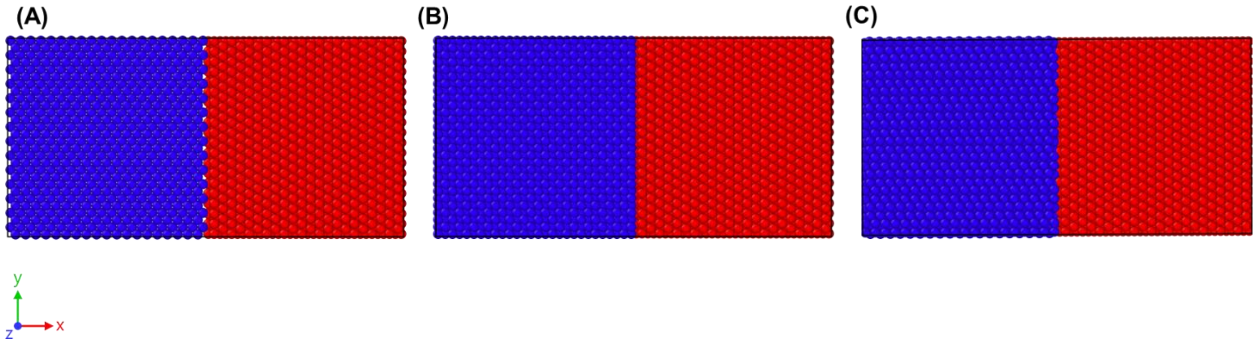

Figure S7. Simulation models with two same-size single-crystal-plane Cu blocks: (A) Cu(220)/Cu(111) interface, (B) Cu(200)/Cu(111) interface, (C) Cu(311)/Cu(111) interface.

9. Temperature profile of MD simulation and additional trajectory snapshots.

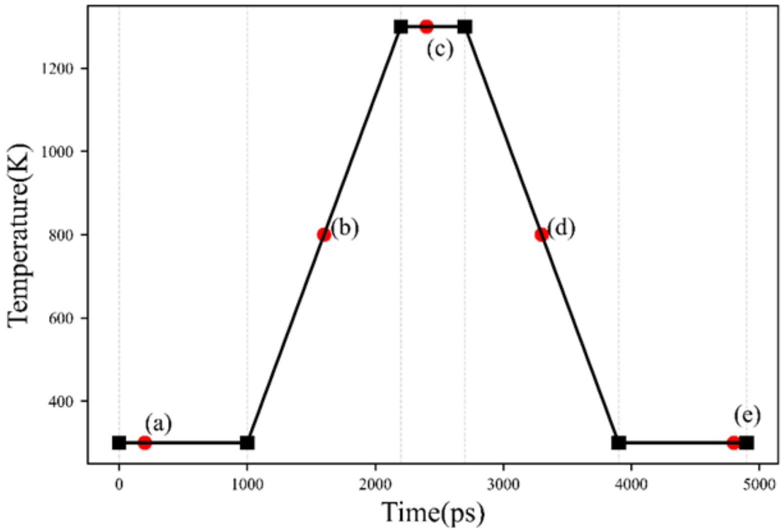

Figure S8. Temperature profile of MD simulation over time. The red dots represent the time spot where the trajectory snapshots were taken. (a) 300 ps; (b) 1600 ps; (c) 2400 ps; (d) 3300 ps; (e) 4800 ps.

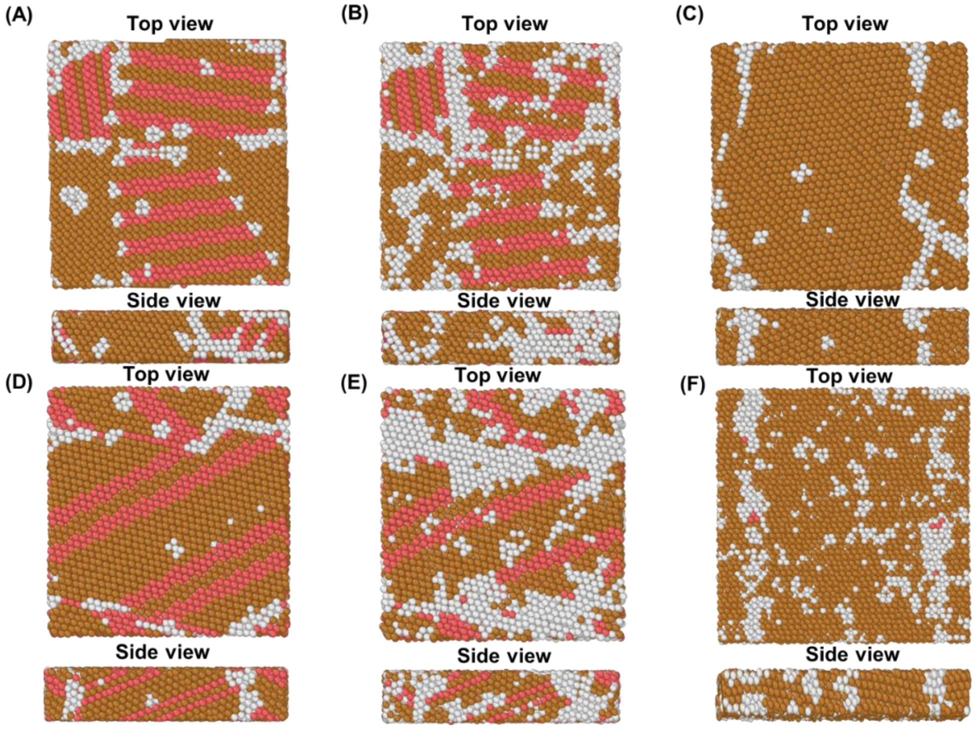

Figure S9. Additional trajectory snapshots of MD simulations of the annealing process: Top and side views of 3×3 models (A–C) with and (D–F) without Cu(111) seed block before, during and after annealing. (A) State of model with Cu(111) seed block after annealing at 300 K and 300 ps. (B) State of model with Cu(111) seed block after annealing at 800 K and 1600 ps. (C) State of model with Cu(111) seed block after annealing at 800 K for 3300 ps. (D) State of model without Cu(111) seed block after annealing at 300 K and 300 ps. (E) State of model without Cu(111) seed block after annealing at 800 K and 1600 ps. (f) State of model with Cu(111) seed block after annealing at 800 K for 3300 ps. Brown color represents face-centered-cubic (fcc) structure, and white color represents stacking fault displacement; and the pink color represents hexagonal close-packed (hcp) structure.

10. Annealing Cu foils in the presence of residual air in the CVD chamber

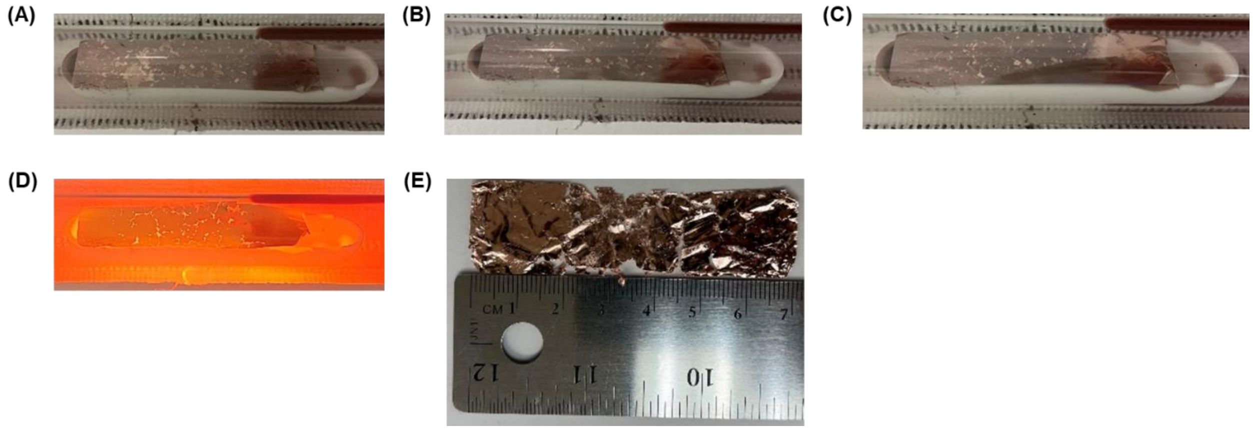

Figure S10. Photographs of Cu foils annealed in the presence of residual air at 1060 °C for (A) 1 h, (B) 2 h, or (C) 3 h. Photos were taken after cooling the samples in the quartz tube to about 500 °C. (D) Cu foil annealed at 1060 °C for about 3.5 h. Photo was taken after opening the methane gauge to the quartz tube and cooling to about 900 °C. (E) Cu foil in (D) after cooling to room temperature.

11. Temperature profile of the furnace during graphene growth

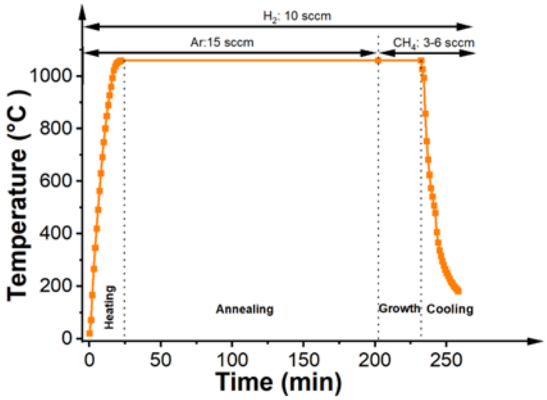

Figure S11. Temperature-time profile of the furnace during the CVD growth of graphene.

12. Optimization of laser power and irradiation time for Raman spectroscopy

Raman spectra were collected at laser power of 0.2 mW or 2 mW for 1 s, 5 s, or 10 s, respectively. At 0.2 mW, the noise level was high in the Raman spectra (**Fig. S12A**), giving the signal-to-noise (*S/N*) ratio of 2.1, 5.4 and 10 for 1 s, 5 s, or 10 s irradiation, respectively (**Fig. S12B**). At 2 mW laser power, the noise level decreased substantially (**Fig. S12A**). The *S/N* was higher for the spectrum collected at 2 mW for 1 s (*S/N*=23) than 0.2 mW for 10 s (*S/N*=12), indicating that the laser power is more important in reducing spectral noise than the irradiation time. The laser power of 2 mW for 5 s and 10 s gave significantly higher *S/N* of 52 and 93, respectively (**Fig. S12B**). Additionally, we irradiated graphene repeatedly at the same loaction at 2 mW six times at 5 s each for a total of 30 s. No obvious increase in I_D/I_G was observed (**Fig. S12D**). In fact, there was no correlation between I_D/I_G and the irradiation time (**Fig. S12C**), demonstrating that no defects were generated in graphene after irradiating at 2 mW for 30 s.

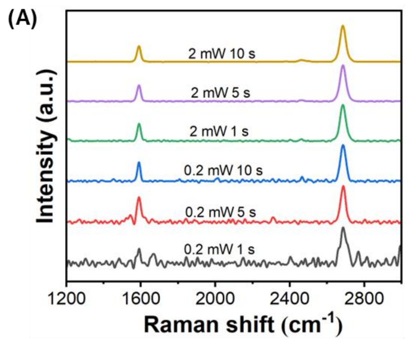

(B)

Irradiation conditions (power, time)	ω_D (cm^{-1})	ω_G (cm^{-1})	ω_{2D} (cm^{-1})	FWHM$_G$ (cm^{-1})	FWHM$_{2D}$ (cm^{-1})	I_{2D}/I_G	I_D/I_G	S/N
0.2 mW, 1 s	N/A	1590.5	2685.5	25.5	26.6	2.34	0.335	2.1
0.2 mW, 5 s	N/A	1590.0	2687.0	24.2	31.3	1.45	0.099	5.4
0.2 mW, 10 s	N/A	1590.0	2685.5	19.2	35.1	1.91	0.042	12
2 mW, 1 s	N/A	1590.5	2684.5	21.4	36.4	2.10	0.025	23
2 mW, 5 s	N/A	1590.0	2685.0	23.3	36.4	2.25	0.025	52
2 mW, 10 s	N/A	1590.0	2684.5	23.2	36.2	2.30	0.011	93

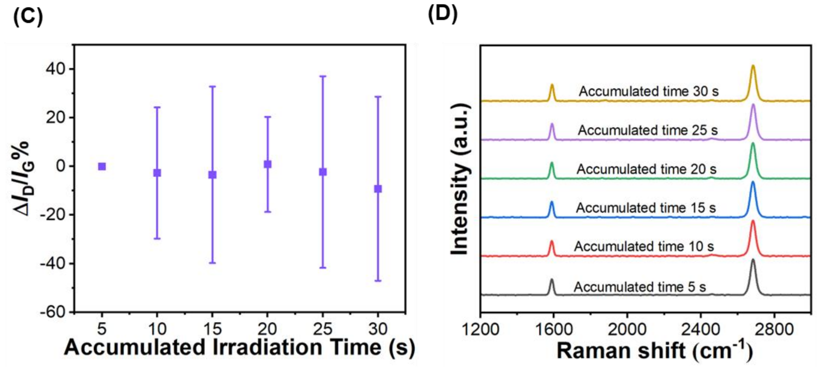

Figure S12. (A) Raman spectra of graphene collected under different laser power and irradiation time. (B) Summary of data in (A). ω_D, ω_G and ω_{2D} are wavenumbers of the D peak, G peak, and 2D peak, respectively. FWHM$_G$ and FWHM$_{2D}$ are the full width at half maximum of G peak and 2D peak, respectively. I_{2D}/I_G and I_D/I_G are intensity ratios of 2D peak vs. G peak, and D peak vs. G peaks, respectively. The signal-to-noise (*S/N*) ratio was calculated using the formula: $S/N = 2H/h$, where H is the height of the G peak, and h is the maximum fluctuation of the background noise, collected over a distance of 20 times the width at half-eight of the G peak.[10] (C) Percent change in I_D/I_G ($\Delta I_D/I_G\% = (I_D/I_G-(I_D/I_G)_o)/(I_D/I_G)_o \times 100\%$) vs. accumulated irradiation time collected from 5 different spots on the sample. (D) A representative Raman spectrum of graphene irradiated at 2 mW laser power for 5 s each and six times on the same spot for a total of 30 s in (C). N/A: D peak intensity is below the noise level.

13. Optimization of annealing conditions for the growth of monolayer graphene.

(A)

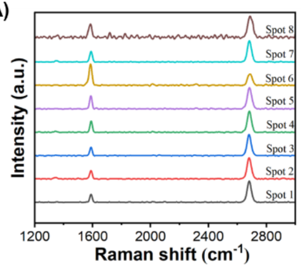

(B)

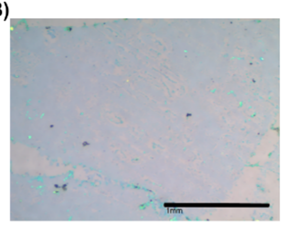

(C)

Spot	ω_D (cm^{-1})	ω_G (cm^{-1})	ω_{2D} (cm^{-1})	FWHM$_G$ (cm^{-1})	FWHM$_{2D}$ (cm^{-1})	I_{2D}/I_G	I_D/I_G
1	N/A	1589.5	2681.5	19.9	33.1	2.70	0.025
2	1345.5	1589.0	2680.5	21.2	34.0	2.57	0.176
3	N/A	1589.0	2680.0	20.2	32.5	2.55	0.051
4	N/A	1590.0	2682.0	19.2	32.6	1.89	0.090
5	N/A	1588.0	2683.0	19.9	34.4	1.64	0.018
6	N/A	1586.0	2687.0	23.4	39.5	0.53	0.019
7	1349.0	1589.5	2682.5	21.9	32.8	2.03	0.114
8	N/A	1585.5	2687.0	28.2	41.6	1.61	0.154
Average	1347.3	1588.3	2682.9	21.7	35.1	1.94	0.081
S.D.	1.75	1.58	2.52	2.74	3.28	0.67	0.059

(D)

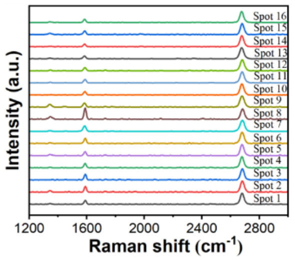

(E)

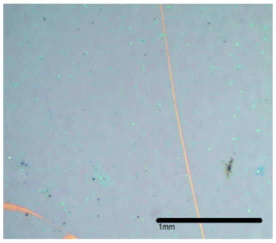

(F)

Spot	ω_D (cm^{-1})	ω_G (cm^{-1})	ω_{2D} (cm^{-1})	FWHM$_G$ (cm^{-1})	FWHM$_{2D}$ (cm^{-1})	I_{2D}/I_G	I_D/I_G
1	1345.0	1590.0	2679.5	18.7	30.2	2.90	0.166
2	1344.5	1590.5	2681.0	18.1	30.6	1.97	0.241
3	N/A	1590.5	2681.5	18.1	30.1	1.98	0.065
4	N/A	1589.0	2678.5	18.4	28.8	2.52	0.088
5	1344.5	1589.0	2679.5	19.7	30.2	2.82	0.251
6	N/A	1590.0	2679.5	19.4	31.8	2.18	0.114
7	1344.0	1585.5	2682.0	23.7	35.8	2.01	0.106
8	1347.0	1589.0	2682.5	18.5	30.6	0.91	0.222
9	1343.0	1584.5	2679.0	23.3	35.2	2.80	0.660
10	N/A	1587.5	2678.0	21.1	30.3	3.32	0.084
11	1348.5	1588.5	2680.0	21.7	32.5	3.24	0.146
12	1346.5	1588.5	2681.0	21.4	31.9	3.46	0.244
13	1343.5	1584.5	2676.5	22.0	33.6	4.12	0.233
14	N/A	1585.5	2677.5	20.3	30.3	4.30	0.158
15	1343.5	1586.5	2679.5	21.5	29.8	3.04	0.341
16	1343.0	1585.0	2676.5	20.4	28.8	3.44	0.191
Average	1344.8	1587.8	2679.5	20.4	31.3	2.81	0.207
S.D.	1.71	2.11	1.76	1.75	2.01	0.84	0.138

(G)

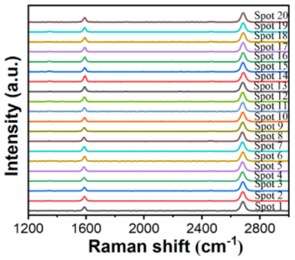

(H)

Spot	ω_D (cm^{-1})	ω_G (cm^{-1})	ω_{2D} (cm^{-1})	FWHM$_G$ (cm^{-1})	FWHM$_{2D}$ (cm^{-1})	I_{2D}/I_G	I_D/I_G
1	1342.5	1588.0	2682.5	22.8	35.4	2.42	0.075
2	N/A	1588.0	2682.0	23.9	36.4	2.14	0.038
3	1344.5	1587.5	2682.5	23.0	35.7	2.60	0.096
4	1344.5	1585.5	2679.5	23.0	36.7	2.65	0.106
5	N/A	1585.0	2679.5	23.0	35.4	2.17	0.035
6	1344.5	1589.0	2683.0	22.6	34.9	1.91	0.082
7	N/A	1587.0	2679.5	22.0	36.6	1.98	0.050
8	1341.0	1585.5	2679.0	22.2	35.0	2.29	0.054
9	1340.5	1587.0	2681.5	22.7	35.0	2.43	0.070
10	N/A	1589.0	2682.0	21.2	35.3	2.38	0.052
11	1343.5	1588.5	2681.5	21.3	34.1	2.39	0.118
12	1347.5	1588.0	2681.0	22.6	34.9	2.02	0.078
13	N/A	1590.0	2682.5	22.4	36.1	1.97	0.030
14	1347.5	1592.0	2686.0	21.6	36.4	1.57	0.111
15	1346.5	1590.0	2683.0	20.8	34.3	2.29	0.141
16	1346.0	1590.0	2684.0	21.5	34.2	1.97	0.106
17	1344.0	1590.5	2684.0	21.5	34.3	2.07	0.097
18	N/A	1591.0	2685.0	21.5	34.9	2.12	0.032
19	1345.5	1590.0	2684.0	21.4	34.3	2.13	0.128
20	1347.5	1590.0	2684.5	22.3	35.2	2.10	0.024
Average	1344.7	1588.6	2682.3	22.2	35.3	2.18	0.076
S.D.	2.19	1.88	1.91	0.77	0.81	0.25	0.035

(I)

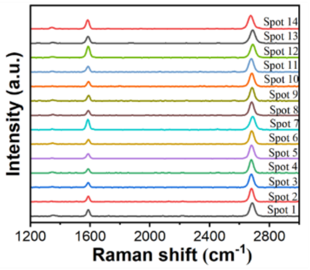

(J)

Spot	ω_D (cm^{-1})	ω_G (cm^{-1})	ω_{2D} (cm^{-1})	FWHM$_G$ (cm^{-1})	FWHM$_{2D}$ (cm^{-1})	I_{2D}/I_G	I_D/I_G
1	1352.5	1587.5	2685.5	22.2	37.8	2.04	0.153
2	N/A	1587.5	2680.0	20.9	32.3	2.43	0.082
3	1346.5	1587.0	2679.0	22.8	35.9	2.84	0.116
4	1346.0	1586.5	2679.0	21.8	33.0	3.19	0.186
5	1344.0	1586.5	2680.5	23.9	36.7	2.77	0.093
6	1348.5	1588.5	2681.5	22.4	34.9	2.95	0.205
7	1345.5	1585.0	2688.0	24.2	40.7	1.30	0.048
8	1350.5	1587.5	2681.5	22.2	36.0	2.77	0.179
9	1348.5	1589.5	2684.5	22.8	34.5	2.46	0.165
10	N/A	1589.0	2682.5	23.5	36.5	2.80	0.052
11	1347.0	1586.5	2679.0	24.8	39.0	2.37	0.133
12	1348.0	1587.0	2688.5	26.7	39.0	1.17	0.071
13	1349.0	1586.0	2687.5	26.4	41.2	1.92	0.116
14	1343.5	1583.5	2675.0	26.1	40.8	1.50	0.148
Average	1347.5	1587.0	2682.3	23.6	37.0	2.32	0.125
S.D.	2.49	1.49	3.86	1.75	2.74	0.62	0.049

(K)

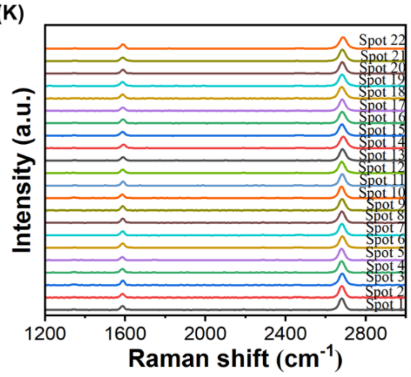

(L)

Spot	ω_D (cm^{-1})	ω_G (cm^{-1})	ω_{2D} (cm^{-1})	FWHM$_G$ (cm^{-1})	FWHM$_{2D}$ (cm^{-1})	I_{2D}/I_G	I_D/I_G
1	N/A	1587.0	2680.0	22.5	32.4	2.83	0.110
2	1347.0	1587.5	2680.5	23.1	32.5	3.08	0.101
3	1344.0	1588.5	2682.0	20.9	36.3	2.51	0.150
4	1345.5	1586.0	2678.5	24.2	34.9	3.12	0.155
5	1344.0	1585.0	2678.5	22.3	33.9	3.65	0.100
6	1352.0	1588.0	2683.0	25.0	35.5	2.61	0.052
7	N/A	1587.5	2680.5	23.8	35.7	2.85	0.032
8	N/A	1588.0	2681.0	22.4	35.5	2.62	0.015
9	N/A	1588.0	2681.0	22.3	35.1	2.68	0.041
10	1345.0	1585.0	2678.5	22.1	33.0	3.24	0.170
11	1350.0	1589.5	2682.0	20.9	33.5	2.86	0.076
12	N/A	1587.5	2680.0	24.5	35.6	3.28	0.063
13	1347.5	1589.0	2683.0	25.2	36.1	3.37	0.138
14	N/A	1592.0	2687.0	25.7	40.9	3.23	0.082
15	1347.5	1587.0	2680.5	26.8	38.3	3.01	0.098
16	1345.5	1589.5	2681.5	24.8	39.1	2.95	0.113
17	N/A	1588.5	2681.5	25.4	34.8	3.47	0.042
18	1340.0	1587.0	2679.5	24.1	39.1	2.90	0.084
19	N/A	1586.5	2681.0	26.9	37.9	2.92	0.064
20	1344.0	1588.5	2682.0	22.9	34.8	2.96	0.108
21	1347.0	1588.0	2682.5	28.5	37.0	3.30	0.152
22	N/A	1589.0	2686.0	25.3	41.7	2.60	0.076
Average	1346.1	1587.8	2681.4	24.1	36.1	3.00	0.092
S.D.	2.88	1.52	2.08	1.93	2.49	0.30	0.043

(M)

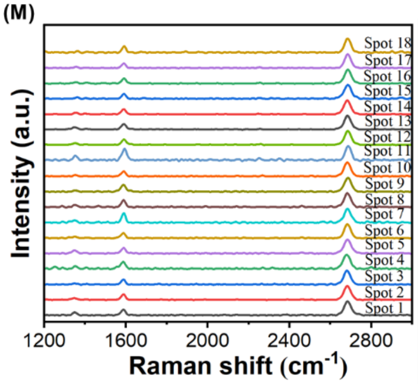

(N)

Spot	ω_D (cm^{-1})	ω_G (cm^{-1})	ω_{2D} (cm^{-1})	FWHM$_G$ (cm^{-1})	FWHM$_{2D}$ (cm^{-1})	I_{2D}/I_G	I_D/I_G
1	1349.0	1587.5	2683.5	25.9	37.8	2.41	0.521
2	1347.0	1588.0	2683.0	25.3	40.3	2.52	0.303
3	1347.5	1588.0	2681.0	24.5	38.0	2.84	0.193
4	1351.5	1586.5	2680.0	29.1	42.3	2.02	0.365
5	1348.0	1589.0	2683.5	23.5	40.4	2.50	0.547
6	1350.0	1587.5	2683.0	26.1	37.8	2.77	0.326
7	1349.0	1589.5	2683.0	22.8	37.4	1.47	0.233
8	1349.5	1588.5	2682.5	25.2	38.1	2.04	0.301
9	1350.0	1588.0	2686.5	25.6	40.0	2.00	0.186
10	N/A	1589.5	2683.0	26.8	38.6	2.86	0.235
11	1351.5	1593.5	2686.5	28.2	35.9	1.23	0.388
12	1350.0	1590.5	2685.0	25.1	37.6	2.62	0.305
13	1348.0	1590.0	2683.5	26.4	38.2	2.56	0.405
14	1354.0	1589.0	2682.5	25.2	39.5	2.70	0.280
15	1363.0	1590.5	2685.0	24.8	37.1	2.93	0.297
16	1356.0	1591.0	2685.5	24.7	38.4	2.75	0.251
17	1356.0	1590.5	2684.5	25.3	36.4	3.07	0.274
18	N/A	1591.5	2683.5	23.6	36.9	2.27	0.233
Average	1351.3	1589.4	2683.6	25.4	38.4	2.42	0.314
S.D.	4.05	1.67	1.65	1.50	1.55	0.49	0.098

Figure S13. (A) Raman spectra and (B) optical microscopy image of graphene grown on as-reveived polycrystalline Cu foil annealed at 1000 °C for 1 h and subsequently transferred to a silicon wafer. (C) Summary of Raman data in (A). (D) Raman spectra and (E) optical microscopy image of graphene grown on electropolished Cu foil annealed at 1000 °C for 70 min and subsequently transferred to a silicon wafer. (F) Summary of Raman data in (D). Raman spectra and summary of Raman data of graphene grown on electropolished Cu foil annealed at (G, H) 1040 °C for 3 h, (I, J) 1050 °C for 3 h, (K, L) 1060 °C for 2 h, (M, N) 1060 °C for 17 h. All Raman spectra were collected with the laser power of 2 mW and irradiation time of 5 s.

14. Summary of the Raman data and characterization of two additional batches of graphene grown on electropolished Cu foil annealed at 1060 °C for 3 h.

Table S2. Summary of the Raman data on graphene grown on electropolished Cu foil annealed at 1060 °C for 3 h. The data are taken from **Figure 7B**.

Spot	ω_D (cm^{-1})	ω_G (cm^{-1})	ω_{2D} (cm^{-1})	$FWHM_G$ (cm^{-1})	$FWHM_{2D}$ (cm^{-1})	I_{2D}/I_G	I_D/I_G
1	N/A	1587.5	2681.0	24.7	39.3	2.99	0.051
2	N/A	1588.0	2681.0	24.3	39.0	3.17	0.047
3	N/A	1590.5	2685.0	22.8	36.7	2.59	0.096
4	N/A	1587.5	2680.5	23.8	45.2	1.52	0.034
5	N/A	1589.5	2683.5	25.4	37.4	2.10	0.121
6	N/A	1587.0	2680.5	22.9	39.7	2.71	0.067
7	N/A	1587.0	2676.5	23.5	39.0	2.30	0.013
8	N/A	1587.0	2683.0	27.3	40.5	3.00	0.060
9	N/A	1588.5	2681.5	23.2	39.0	3.00	0.038
10	N/A	1588.0	2681.0	24.6	38.7	1.49	0.038
11	N/A	1590.0	2685.0	23.4	36.9	1.30	0.027
12	N/A	1590.0	2684.5	23.5	36.5	1.47	0.023
13	N/A	1587.0	2679.0	25.0	45.8	2.27	0.030
14	N/A	1588.5	2681.0	23.4	34.8	3.07	0.062
15	N/A	1587.0	2678.5	21.0	36.0	3.38	0.036
16	N/A	1588.5	2681.0	22.1	36.0	3.50	0.036
17	N/A	1588.5	2682.0	22.3	37.1	3.27	0.037
Average	N/A	1588.2	2681.4	23.7	38.7	2.54	0.048
S.D.	N/A	1.14	2.22	1.41	2.92	0.71	0.026

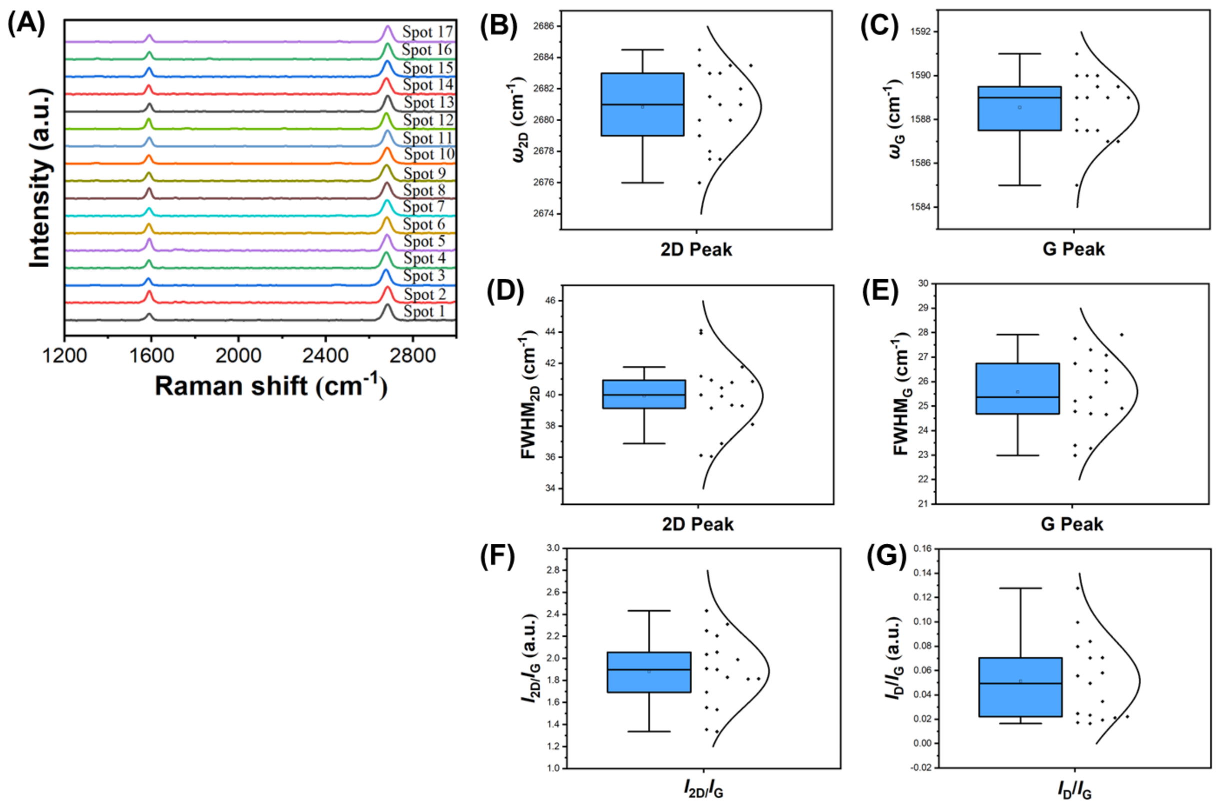

Figure S14. Characterization of graphene grown on electropolished Cu foil annealed at 1060 °C for 3 h: Batch #2. (A) Raman spectra collected at 17 different locations on graphene randomly selected from two samples. Each sample was about 0.5 cm × 0.5 cm in size. Spectra were collected using the laser power of 2 mW and irradiation time of 5 s. (B-G) Boxplots of all Raman spectroscopy data in (A).

Table S3. Summary of Raman data taken from **Fig. S14A**.

Spot	ω_D (cm^{-1})	ω_G (cm^{-1})	ω_{2D} (cm^{-1})	FWHM$_G$ (cm^{-1})	FWHM$_{2D}$ (cm^{-1})	I_{2D}/I_G	I_D/I_G
1	N/A	1589.0	2683.5	27.8	40.0	2.43	0.080
2	N/A	1589.5	2683.0	26.7	40.9	1.35	0.023
3	1337.0	1585.0	2676.0	25.4	40.4	2.25	0.056
4	N/A	1588.0	2677.5	23.4	40.8	2.04	0.050
5	N/A	1590.0	2681.0	24.8	39.1	1.34	0.017
6	N/A	1587.5	2680.0	26.5	39.9	1.69	0.035
7	N/A	1589.0	2681.0	27.9	41.8	2.06	0.021
8	N/A	1589.0	2680.0	25.2	41.2	1.55	0.025
9	N/A	1587.5	2679.0	27.3	43.9	1.91	0.070
10	1347.5	1587.5	2681.5	27.1	44.1	1.90	0.100
11	N/A	1589.5	2683.0	26.5	39.3	1.83	0.058
12	N/A	1587.0	2677.5	23.0	36.1	1.54	0.017
13	N/A	1591.0	2683.5	23.3	36.1	1.99	0.019
14	N/A	1587.0	2678.0	24.7	40.9	1.81	0.022
15	1353.5	1589.0	2682.0	26.0	39.3	1.82	0.084
16	N/A	1590.0	2683.5	24.7	36.9	2.21	0.128
17	N/A	1590.0	2684.5	24.9	38.1	2.31	0.071
Average	1346.0	1588.6	2680.9	25.6	39.9	1.88	0.051
S.D.	6.82	1.45	2.48	1.50	2.23	0.31	0.032

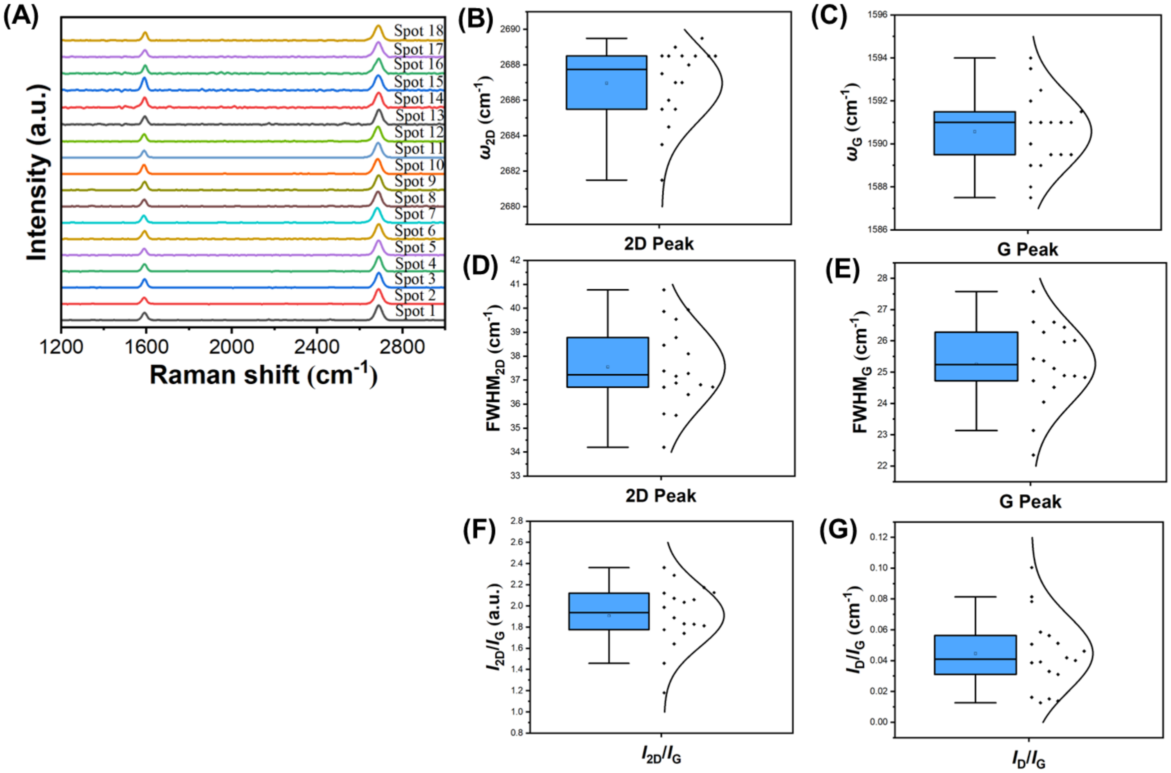

Figure S15. Characterization of graphene grown on electropolished Cu foil annealed at 1060 °C for 3 h: Batch #3. (A) Raman spectra collected at 18 different locations on graphene randomly selected from two samples. Each sample was about 0.5 cm × 0.5 cm in size. Spectra were collected using the laser power of 2 mW and irradiation time of 5 s. (B-G) Boxplots of all Raman spectroscopy data in (A).

Table S4. Summary of Raman data taken from **Fig. S15A.**

Spot	ω_D (cm^{-1})	ω_G (cm^{-1})	ω_{2D} (cm^{-1})	FWHM$_G$ (cm^{-1})	FWHM$_{2D}$ (cm^{-1})	I_{2D}/I_G	I_D/I_G
1	N/A	1591.0	2688.5	26.6	36.7	1.99	0.051
2	N/A	1589.5	2687.5	26.6	37.4	2.36	0.059
3	N/A	1591.0	2689.0	24.9	35.6	1.74	0.056
4	N/A	1591.0	2688.5	25.4	34.2	2.03	0.051
5	N/A	1589.5	2688.0	24.9	37.2	2.29	0.015
6	N/A	1591.0	2688.5	26.4	36.7	1.89	0.039
7	N/A	1587.5	2681.5	25.4	38.8	2.06	0.014
8	N/A	1589.5	2685.5	25.1	37.3	2.17	0.081
9	N/A	1591.0	2688.5	26.3	36.9	1.78	0.078
10	N/A	1588.0	2683.5	24.7	38.5	1.64	0.016
11	N/A	1589.0	2685.5	24.0	36.4	2.12	0.013
12	N/A	1589.0	2684.5	24.5	36.8	2.07	0.039
13	N/A	1592.0	2689.5	27.6	35.5	1.83	0.100
14	N/A	1591.5	2688.5	26.0	38.1	1.46	0.042
15	N/A	1590.0	2686.0	26.0	40.8	1.18	0.040
16	N/A	1594.0	2688.5	22.4	39.9	1.83	0.046
17	N/A	1592.5	2687.0	24.8	39.5	2.13	0.033
18	N/A	1593.5	2687.0	23.1	39.9	1.81	0.031
Average	N/A	1590.6	2687.0	25.3	37.6	1.91	0.045
S.D.	N/A	1.71	2.10	1.25	1.69	0.28	0.024

15. Home-built CVD setup

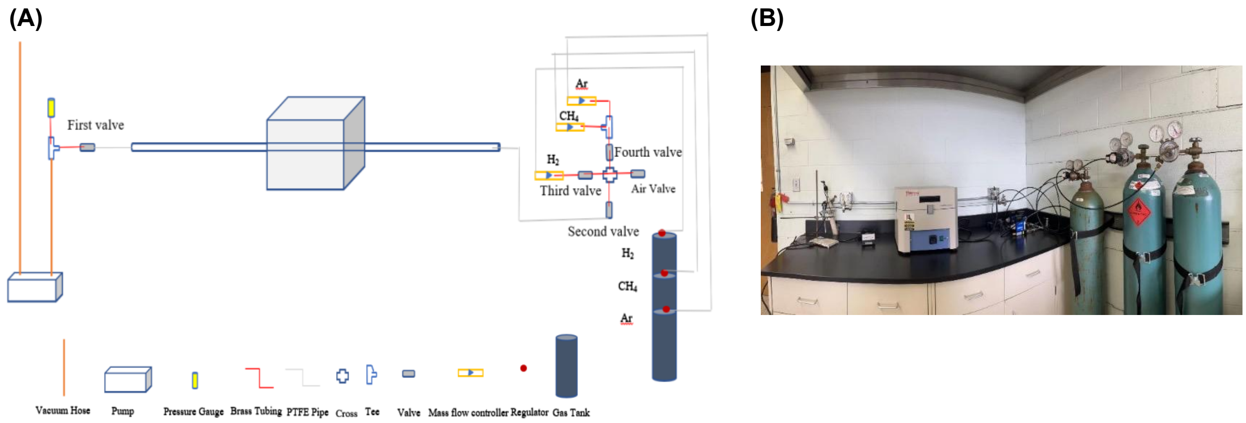

Figure S16. (A) Schematic of the home-built CVD setup. (B) Photograph of the CVD setup. The length of the heating zone of the furnace is 30.5 cm. The heating zone temperature variation is ± 3 ℃ over 8.4 cm. Thus, the heating zone can provide uniform temperatures for annealing.

16. Additional two batches of graphene on Cu(111)

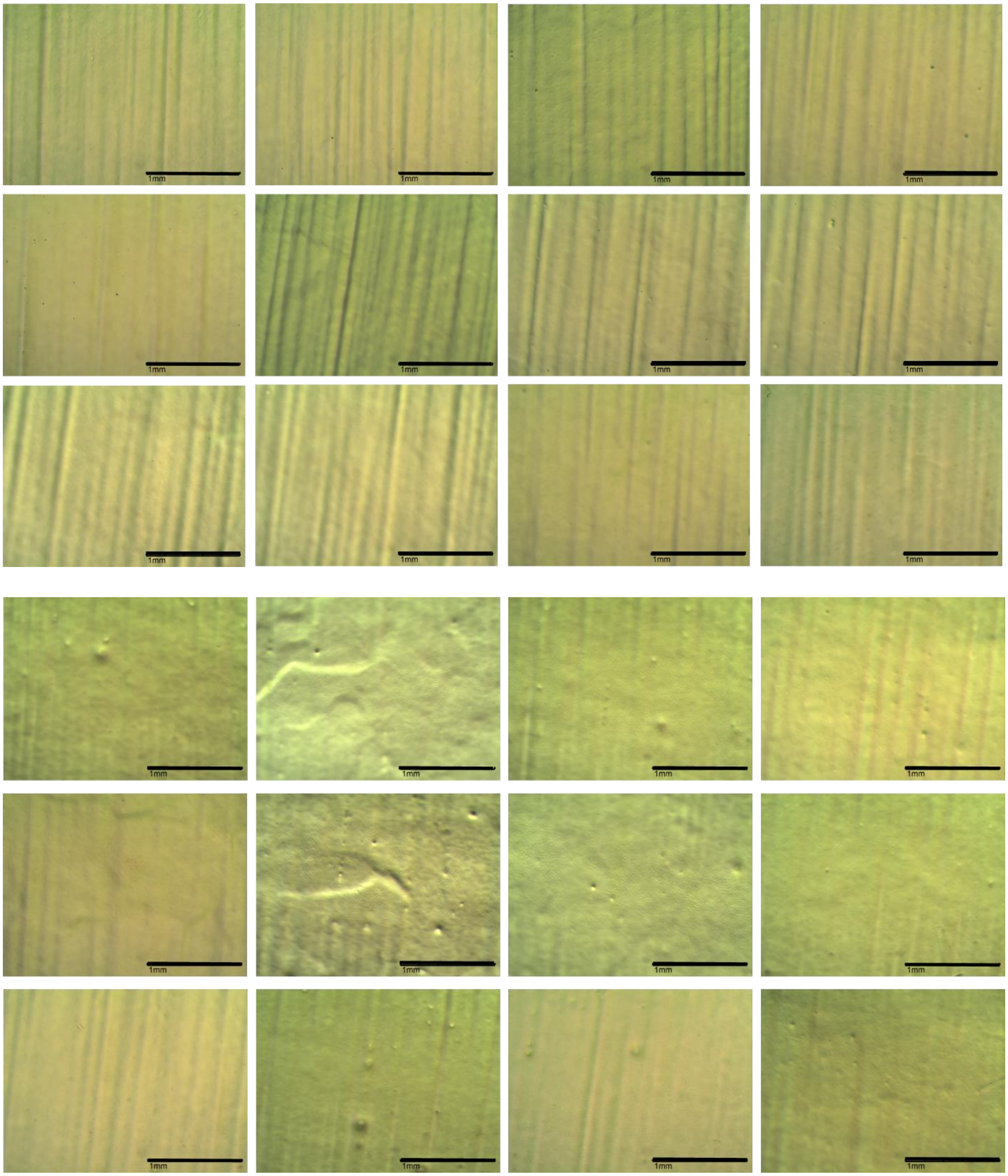

Figure S17. Optical images of additional two batches of graphene on Cu(111). The samples were prepared using the optimized conditions of annealing electropolished Cu foils at 1060 °C for 3 h followed by CVD growth of graphene from methane at 1060 °C for 30 minutes. The images were taken immediately after the samples were removed from the CVD chamber. Scale bars: 1 mm.

References

(1) Brown, L.; Lochocki, E. B.; Avila, J.; Kim, C. J.; Ogawa, Y.; Havener, R. W.; Kim, D. K.; Monkman, E. J.; Shai, D. E.; Wei, H. I.; et al. Polycrystalline graphene with single crystalline electronic structure. *Nano Lett.* **2014**, *14* (10), 5706-5711.
(2) Nguyen, V. L.; Shin, B. G.; Duong, D. L.; Kim, S. T.; Perello, D.; Lim, Y. J.; Yuan, Q. H.; Ding, F.; Jeong, H. Y.; Shin, H. S.; et al. Seamless stitching of graphene domains on polished copper (111) foil. *Adv. Mater.* **2015**, *27* (8), 1376-1382.
(3) Reckinger, N.; Tang, X.; Joucken, F.; Lajaunie, L.; Arenal, R.; Dubois, E.; Hackens, B.; Henrard, L.; Colomer, J. F. Oxidation-assisted graphene heteroepitaxy on copper foil. *Nanoscale* **2016**, *8* (44), 18751-18759.
(4) Jin, S.; Huang, M.; Kwon, Y.; Zhang, L.; Li, B.-W.; Oh, S.; Dong, J.; Luo, D.; Biswal, M.; Cunning, B. V.; et al. Colossal grain growth yields single-crystal metal foils by contact-free annealing. *Science* **2018**, *362* (6418), 1021-1025.
(5) Luo, D.; Wang, M.; Li, Y.; Kim, C.; Yu, K. M.; Kim, Y.; Han, H.; Biswal, M.; Huang, M.; Kwon, Y.; et al. Adlayer-Free Large-Area Single Crystal Graphene Grown on a Cu(111) Foil. *Adv. Mater.* **2019**, *31* (35), e1903615.
(6) Jo, I.; Park, S.; Kim, D.; Moon, J. S.; Park, W. B.; Kim, T. H.; Kang, J. H.; Lee, W.; Kim, Y.; Lee, D. N.; et al. Tension-controlled single-crystallization of copper foils for roll-to-roll synthesis of high-quality graphene films. *2D Materials* **2018**, *5* (2), 024002.
(7) Xu, X.; Zhang, Z.; Dong, J.; Yi, D.; Niu, J.; Wu, M.; Lin, L.; Yin, R.; Li, M.; Zhou, J.; et al. Ultrafast epitaxial growth of metre-sized single-crystal graphene on industrial Cu foil. *Sci. Bull.* **2017**, *62* (15), 1074-1080.
(8) Sun, L.; Chen, B.; Wang, W.; Li, Y.; Zeng, X.; Liu, H.; Liang, Y.; Zhao, Z.; Cai, A.; Zhang, R.; et al. Toward Epitaxial Growth of Misorientation-Free Graphene on Cu(111) Foils. *ACS Nano* **2022**, *16* (1), 285-294.
(9) Chen, H.; Liu, X.; Huang, Y.; Li, G.; Yu, F.; Xiong, F.; Zhang, M.; Sun, L.; Yang, Q.; Jia, K.; et al. Oxidization-Temperature-Triggered Rapid Preparation of Large-Area Single-Crystal Cu(111) Foil. *Adv. Mater.* **2023**, *35* (18), e2209755.
(10) Cantu, L. M. L.; Gallo, E. C. A. Explosives and warfare agents remote Raman detection on realistic background samples. *Eur. Phys. J. Plus* **2022**, *137* (2), 207.
